# Histone deacetylase inhibitors improve antisense-mediated exon-skipping efficacy in *mdx* mice

**DOI:** 10.1016/j.omtn.2022.11.017

**Published:** 2022-11-21

**Authors:** Flavien Bizot, Remko Goossens, Thomas Tensorer, Sergei Dmitriev, Luis Garcia, Annemieke Aartsma-Rus, Pietro Spitali, Aurélie Goyenvalle

**Affiliations:** 1 Université Paris-Saclay, UVSQ, Inserm, END-ICAP, 78000 Versailles, France; 2 Department of Human Genetics, Leiden University Medical Center, 2333 ZA Leiden, the Netherlands; 3 SQY Therapeutics, UVSQ, END-ICAP, 78180 Montigny le Bretonneux, France

**Keywords:** MT: antisense oligonucleotides, exon skipping, RNA, transcript imbalance, histone deacetylase inhibitors, Duchenne muscular dystrophy, valproic acid

## Abstract

Antisense-mediated exon skipping is one of the most promising therapeutic strategies for Duchenne muscular dystrophy (DMD), and some antisense oligonucleotide (ASO) drugs have already been approved by the US FDA despite their low efficacy. The potential of this therapy is still limited by several challenges, including the reduced expression of the dystrophin transcript and the strong 5′-3′ imbalance in mutated transcripts. We therefore hypothesize that increasing histone acetylation using histone deacetylase inhibitors (HDACi) could correct the transcript imbalance, offering more available pre-mRNA target and ultimately increasing dystrophin rescue. Here, we evaluated the impact of such a combined therapy on the *Dmd* transcript imbalance phenomenon and on dystrophin restoration levels in *mdx* mice. Analysis of the *Dmd* transcript levels at different exon-exon junctions revealed a tendency to correct the 5′-3′ imbalance phenomenon following treatment with HDACi. Significantly higher levels of dystrophin restoration (up to 74% increase) were obtained with givinostat and valproic acid compared with mice treated with ASO alone. Additionally, we demonstrate an increase in H3K9 acetylation in human myocytes after treatment with valproic acid. These findings indicate that HDACi can improve the therapeutic potential of exon-skipping approaches, offering promising perspectives for the treatment of DMD.

## Graphical abstract

**Figure:**
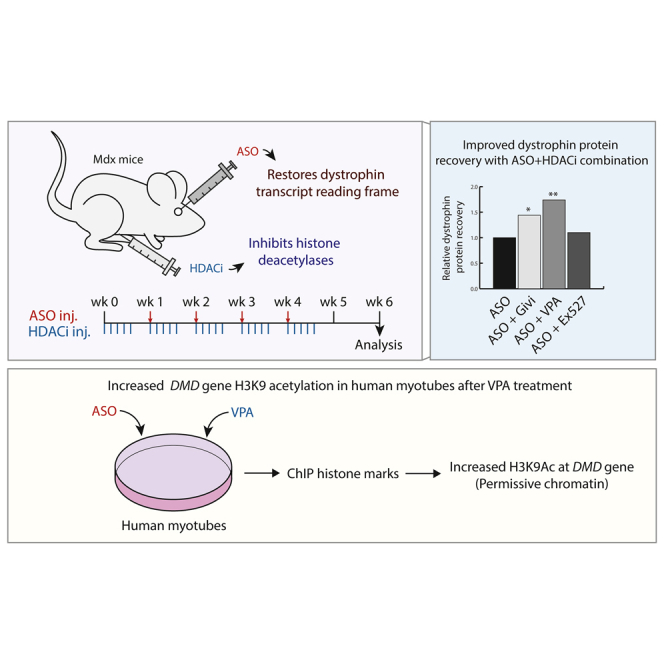

## Teaser

Antisense-mediated exon skipping is one of the most promising therapeutic strategies for Duchenne muscular dystrophy, but its potential is limited by the reduced expression of *Dmd* transcripts. This study shows that histone deacetylase inhibitors can enhance exon-skipping efficacy by increasing *Dmd* transcript levels and can ultimately improve dystrophin rescue in *mdx* mice.

## Introduction

Duchenne muscular dystrophy (DMD) is a genetic, X-linked, muscle-wasting disease caused by different types of mutations in the *DMD* gene, which mostly disrupt the open reading frame and thus lead to an absence of functional dystrophin protein. Becker muscular dystrophy (BMD), which is also caused by mutations in the *DMD* gene, results in a variable but milder phenotype. In contrast to DMD mutations, BMD deletions do not disrupt the open reading frame, allowing translation of a partially truncated but functional dystrophin. Antisense-mediated exon skipping for DMD aims to eliminate one or several exons from the mRNA, by masking key splicing sites with antisense oligonucleotides (ASOs) during the pre-mRNA splicing process. The resulting mRNA will have a restored reading frame and therefore allow the expression of a BMD-like dystrophin. Several ASO-based exon-skipping drugs have already been approved by the US Food and Drug Administration for the treatment of DMD (eteplirsen, golodirsen, viltolarsen, and casimersen targeting exons 51, 53, 53, and 45).^1^ Approval was based on safety and increased dystrophin expression in muscle biopsies of treated patients at relatively low levels (up to ∼5.9% following exon 53 skipping).^2^ Recent studies suggest delayed loss of ambulation and pulmonary decline following long-term eteplirsen treatment compared with a natural history cohort,^3^ and placebo-controlled clinical trials are still ongoing to assess functional outcomes for each of the compounds. However, the efficacy of exon-skipping strategies is still limited by several challenges, leaving much room for improvement.

One of the most recognized challenges of ASO-mediated exon skipping is the delivery of ASOs to the target tissues.^4^^,^^5^ Many efforts are focusing on improving this delivery, notably through the development of alternative chemistries or various conjugates.^6^^,^^7^^,^^8^ Among these, some of us have been working with one particular chemistry named tricyclo-DNA (tcDNA), and we recently demonstrated that conjugation of palmitic acid with tcDNA (palm-tcDNA) significantly enhances their therapeutic potential.^9^ Many of these newly developed compounds are currently or soon to be evaluated in clinical trials. However, another less recognized challenge limits the effect of ASOs independently of their delivery properties: the limited amount of target mRNA. Indeed, transcriptional studies have shown that dystrophin mRNA levels are reduced in muscle when a mutation is present.^10^^,^^11^ This reduction was thought to be caused by nonsense-mediated decay (NMD), which breaks down transcripts with premature termination codons (PTCs) due to nonsense mutations or frameshifts.^12^ However, our recent work has shown that inhibition of NMD similarly impacts wild-type (WT) and dystrophic DMD transcript levels, suggesting that NMD does not specifically affect mutated DMD transcripts.^13^ More importantly, this work confirmed previous findings describing a strong 5′-3′ transcript imbalance in mutated transcripts.^13^^,^^14^ Reduced transcript accumulation toward the 3′ end was also observed in healthy controls,^15^ suggesting that the locus is transcriptionally challenging. This is not surprising given that the *DMD* gene is one of the largest genes in the human genome, spanning 2.2 Mb. However, PTC-containing transcripts appear to be even more affected. The reduced transcriptional output at the DMD locus in the presence of PTC appeared to be at least partly due to a chromatin conformation less prone to transcription in *mdx* mice compared with WT mice. Indeed we previously showed an increased expression of histone methyltransferases, which are responsible for the methylation of lysine 9 on histone 3 (H3K9me3). This increase was mirrored by elevated levels of H3K9me3 in *mdx* mouse muscle compared with healthy controls and an increase of H3K9me3 modification along the *Dmd* locus.^13^ H3K9 methylation is a transcriptionally repressive mark that competes with the transcriptional permissive H3K9 acetylation. Hence, promoting permissive chromatin conformation via, for example, H3K9 acetylation could counteract the effects of increased H3K9 methylation observed in the presence of PTC and increase dystrophin pre-mRNA availability. We hypothesize here that increasing histone acetylation (and therefore decreasing histone methylation) using histone deacetylase inhibitors (HDACi) could correct the transcript imbalance, offering more available pre-mRNA target for exon-skipping approaches.

HDACi have previously been studied in the context of DMD models but not for their effect on dystrophin transcript synthesis. Pharmacological blockade of histone deacetylases (HDACs) has been shown to decrease fibrosis and promote compensatory regeneration in the *mdx* skeletal muscle through follistatin upregulation.^16^^,^^17^^,^^18^ These studies have led to the clinical evaluation of the HDACi givinostat, which is currently in a phase III clinical trial for DMD treatment (www.clinicaltrials.gov, clinical trial identifier NCT02851797). Positive topline data from this phase III trial were announced at the Annual PPMD Conference on June 25^th^, 2022, showing the beneficial effect of givinostat in DMD patients (https://www.businesswire.com/news/home/20220625005001/en/Italfarmaco-Group-Announces-Positive-Topline-Data-from-Phase-3-Trial-Showing-Beneficial-Effect-of-Givinostat-in-Patients-with-Duchenne-Muscular-Dystrophy). We hypothesize that on top of these beneficial effects, HDACi could significantly improve the effect of exon-skipping strategies by increasing the level of *Dmd* transcript. In this study, we have evaluated the impact of such a combined therapy on the *Dmd* transcript imbalance phenomenon and on dystrophin restoration levels both *in vivo* in *mdx* mice and *in vitro* in human myocytes. Adult *mdx* mice were treated with different HDACi: givinostat (a pan HDACi), valproic acid (VPA; inhibitor of class I/II), or EX527 (inhibitor of class III), and also received ASOs aimed at skipping exon 23. Mice receiving the combined therapy with givinostat and VPA showed significantly higher levels of dystrophin restoration compared with mice treated with ASO alone. Additionally, we demonstrate increased H3K9 acetylation along the DMD locus in human myocytes.

## Results

### Effect of HDAC inhibitors on *Dmd* mRNA levels in *mdx* mice

**Figure 1:**
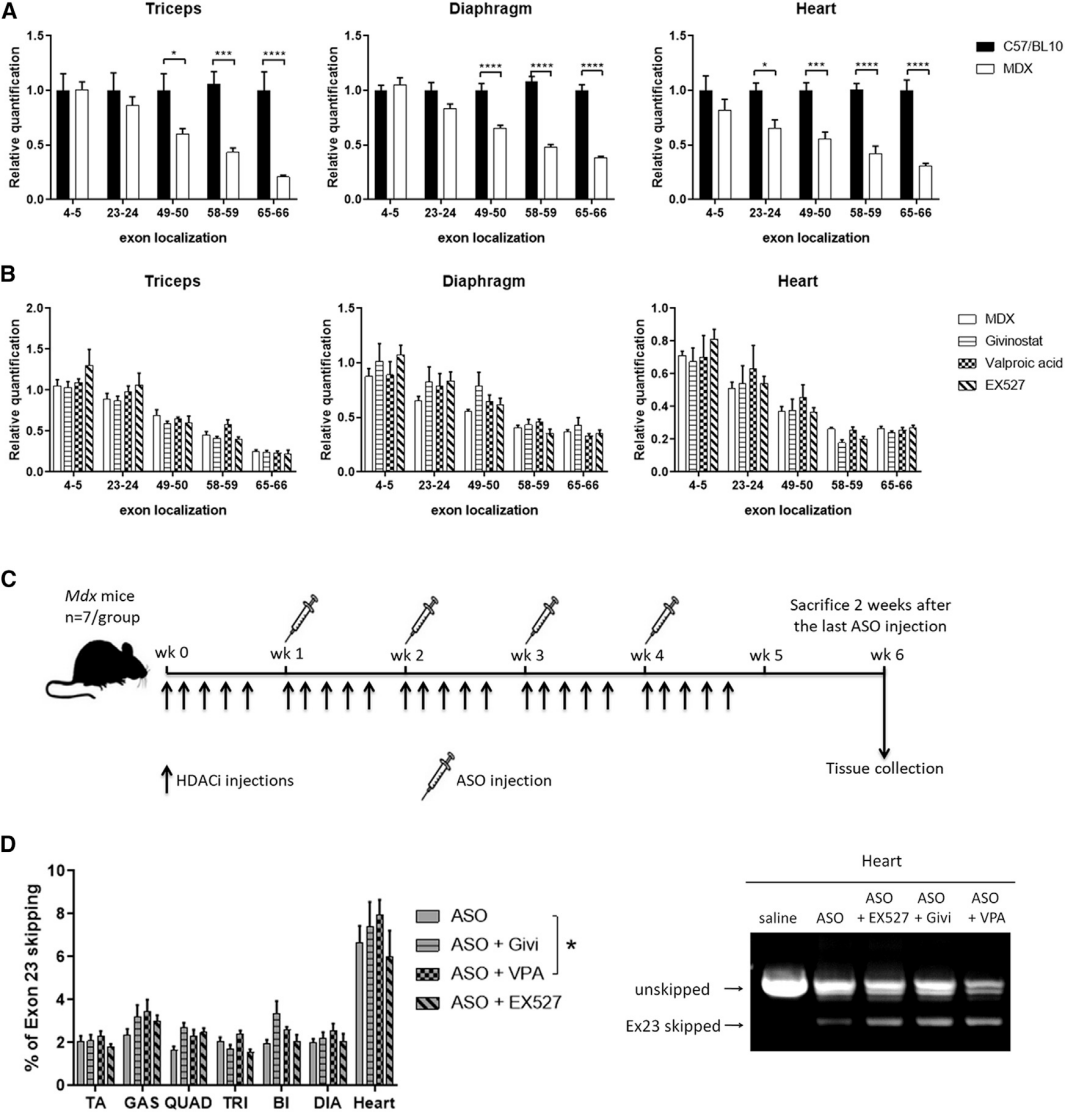
Effect of HDACi on *Dmd* transcript levels and exon-skipping efficacy in *mdx* mice (A) Relative expression of *Dmd* transcript levels obtained in triceps, diaphragm, and heart for several exon-exon junctions along the *Dmd* gene in *mdx* and C57Bl10 mice (n = 6 for C57Bl10 and n = 9 for *mdx* mice) (p = 0.0099 in triceps, p < 0.0001 in diaphragm, and p = 0.0006 in heart between *mdx* and C57Bl10 analyzed by two-way ANOVA). (B) Effect of HDACi treatment on *Dmd* transcript imbalance in triceps, diaphragm, and heart (n = 7 mice per group). No statistical difference was detected overall between HDACi-treated mice and saline-treated mice (p = 0.6694 in triceps, p = 0.5552 in diaphragm, and p = 0.8554 in heart analyzed by two-way ANOVA). (C) Schematic representation of the *mdx* treatment with ASO combined with the various HDACi. (D) Effect of HDACi on exon-skipping level. Left: qPCR quantification of exon 23 using TaqMan qPCR in the different muscle tissues: tibialis anterior (TA), gastrocnemius (GAS), quadriceps (QUAD), triceps (TRI), biceps (BI), diaphragm (DIA), and heart. n = 7 mice per group, ∗p < 0.05 between ASO treatment and ASO + VPA treatment using a two-way ANOVA to compare the two groups. Right: example of the visualization of exon 23 skipping on gel in the heart with one representative mouse for each group (1/7). PCR amplifications between exons 20 and 26 are loaded in a 1.5% agarose gel. The top band corresponds to the unskipped transcript and the lower band to the exon 23 skipped transit. All data are plotted as means ± SEM.

We first studied *Dmd* gene expression at several exon-exon junctions in skeletal and cardiac muscles of 3-month-old *mdx* mice and their WT controls (C57/BL10 mice). The quantitative PCR (qPCR) data obtained from triceps, diaphragm, and heart revealed amounts of *Dmd* transcripts that were significantly lower in all *mdx* muscles compared with WT controls (p = 0.0099 for triceps, p < 0.0001 for diaphragm, and p = 0.0006 for heart) (Figure 1A). No difference was found at the exon junction 4-5, suggesting that initiation of transcription is not altered in *mdx* mice (triceps 100%, diaphragm 105%, and heart 82% compared with WT expression). However, *Dmd* expression was markedly lower in the distal part of the gene in *mdx* muscles where expression levels at the exon 65-66 junction were only 21% in triceps, 38% in diaphragm, and 31% in heart compared with WT expression. These results confirm the strong 5′-3′ transcript imbalance previously described in *mdx* mice due to the nonsense mutation in exon 23. A 5′-3′ imbalance was also detected in WT mice as shown by the absolute quantification of *Dmd* transcripts (Figure S1A), yet slopes were significantly steeper in *mdx* mice.

To investigate whether this imbalance previously linked to epigenetic effects could be counterbalanced by histone acetylation, we treated *mdx* mice with HDACi for 5 weeks. *Mdx* mice received daily administration of givinostat (10 mg/kg by oral gavage 5 days/week^13^), VPA (500 mg/kg intraperitoneally [i.p.] 5 days/week^19^), EX527 (2 mg/kg i.p. 5 days/week^20^) or saline (i.p. 5 days/week).

The quantification of *Dmd* gene expression at several exon-exon junctions by qPCR in muscles from HDACi-treated mice revealed a tendency for some HDACi to increase *Dmd* expression levels, such as EX527 in the proximal exon junctions (4-5 and 24) for all muscles or givinostat across all exon junctions in the diaphragm. However, when we compared all of the exon junctions per treatment with saline, no significant differences emerged (p = 0.8554 in heart, p = 0.6694 in triceps, and p = 0.5552 in diaphragm) (Figure 1B). The *Dmd* gene expression at several exon-exon junctions can also be represented by the regression line (Figure S1B), allowing better visualization of the overall expression levels.

Given that the single treatment with either HDACi (Figure 1B) or ASOs^14^ did not correct the reduction at the 3′ end, we sought to investigate the potential effect of the combination treatment with HDACi and exon-skipping therapy. Therefore, we combined HDACi and palm-tcDNA ASO treatments, as this ASO could efficiently target the donor splice site of mouse Dmd exon 23.^9^ After a first week of HDACi treatment, *mdx* mice were treated once weekly with 25 mg/kg ASO for a total of 4 weeks as presented in Figure 1C. Two weeks after the last ASO injection, tissues were analyzed and exon 23 skipping levels were quantified by TaqMan qRT-PCR (Figure 1D). Four injections of ASO induced exon 23 skipping levels ranging from 2% to 7% in the different muscles. However, co-treatment with VPA slightly increased the skipping levels (1.26-fold change, p = 0.0478, two-way analysis of variance [ANOVA], ASO versus ASO + VPA) (Table 1) as well as co-treatment with givinostat, though not statistically significant (1.21-fold change, p = 0.1916, two-way ANOVA, ASO versus ASO + givinostat). Exon 23 skipping levels were also visualized on gel after RT-PCR. The right panel of Figure 1D shows the gel for heart samples (n = 1/7 mouse per group on the gel) in which the bands corresponding to the exon 23 skipped transcript are clearly visible in ASO-treated samples as opposed to the saline-treated mice. No exon 23 skipping was detected in samples from *mdx* mice treated with HDACi only (data not shown).

**Table 1:** Exon-skipping fold change following HDACi treatment in *mdx* mice

Exon-skipping fold change	TA	GAS	QUAD	TRI	DIA	HEART	Average
ASO + givistatin	1.00	1.36	1.65	0.83	1.09	1.11	1.21
ASO + VPA	1.11	1.45	1.41	1.17	1.28	1.19	1.26
ASO + EX527	0.87	1.27	1.51	0.76	1.03	0.90	1.01

Fold changes are calculated from the levels of exon 23 skipping quantified by TaqMan qPCR obtained in the muscles co-treated with ASO and HDACi compared with the same muscles treated with ASO alone. TA, tibialis anterior; GAS, gastrocnemius; QUAD, quadriceps; TRI, triceps; DIA, diaphragm.

### Effect of HDAC inhibitors on dystrophin protein levels in *mdx* mice

**Figure 2:**
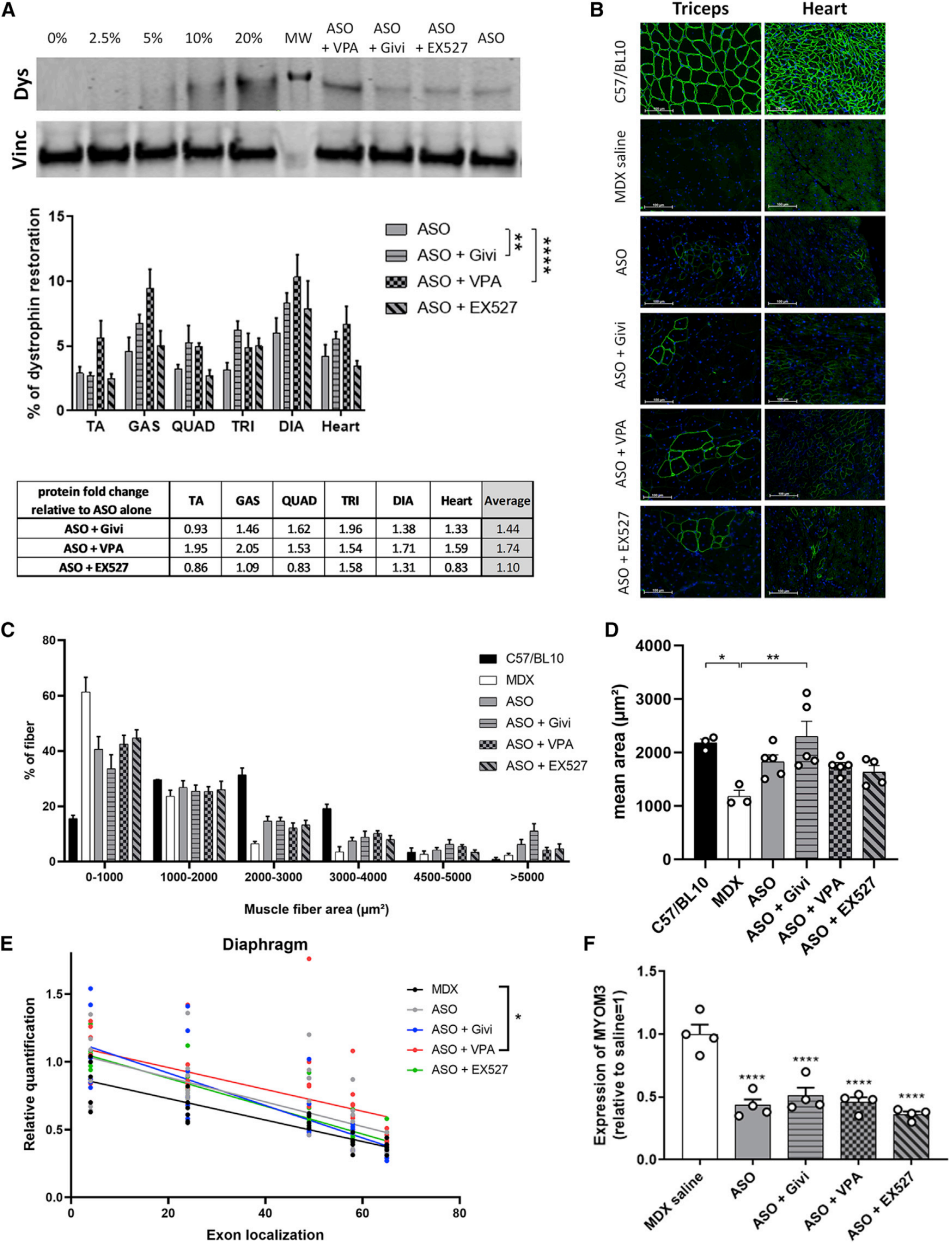
Dystrophin protein expression in *mdx* mice treated with ASO and HDACi (A) Dystrophin restoration in treated *mdx* mice. A typical dystrophin western blot obtained for the heart is shown in the top panel, with vinculin used for normalization. A standard curve made from pooled lysates from C57BL10 (WT) and *mdx* control for each tissue is loaded for quantification (0%, 2.5%, 5%, 10%, and 20% of WT). Middle panel: quantification of dystrophin restoration using Empiria Studio software. n = 7 mice per group, p = 0.0010 between treatments (two-way ANOVA), ∗∗p < 0.01 between ASO alone and ASO + givinostat groups and ∗∗∗∗p < 0.0001 between ASO alone and ASO + VPA groups. p = 0.4496 between ASO and ASO + EX527. The fold changes between protein restoration detected in ASO-treated mice and ASO + HDACi-treated mice is shown in the bottom panel. (B) Detection of dystrophin protein (green staining) by immunostaining on transverse sections of muscle tissues (triceps and heart) from WT and *mdx* mice treated with saline, ASO, or ASO + HDACi. Nuclei are labeled with DAPI (blue staining). Scale bars, 100 μm. (C and D) Graphs representing the distribution (C) and the mean (D) of muscle fiber area in triceps muscles from *mdx* mice treated with ASO or ASO + HDACi and compared with WT (C57/BL10) or saline control *mdx* mice. ∗p < 0.05 and ∗∗p < 0.01 compared with treatment with *mdx* saline. (E) Effect of the combination HDACi + ASO on *Dmd* transcript imbalance in the diaphragm, analyzed by TaqMan qPCR at different exon junctions. n = 7 mice per group; asterisk indicates an adjusted p = 0.0001 between *mdx* and ASO + VPA after multiple testing comparison using Tukey’s method for p-value correction. (F) MYOM3 quantification by western blot in serum of *mdx* mice treated with ASO or ASO + HDACi and compared with saline control *mdx* mice. n = 4 mice/group, ∗∗∗∗p < 0.0001 compared with saline control *mdx* mice. All data are plotted as means ± SEM.

We next assessed the levels of dystrophin expression in the various muscle tissues following treatment with ASO alone or ASO and HDACi. No dystrophin expression was found in samples from *mdx* mice treated with HDACi only (Figure S2A). Significant amounts of dystrophin protein (ranging from 2% to 10%) were detected by western blot in all analyzed tissues after only four administrations of ASO (Figure 2A). We found significantly more dystrophin restoration in *mdx* mice co-treated with VPA (1.74-fold change compared with ASO alone across all muscles, p < 0.0001, two-way ANOVA, ASO versus ASO + VPA) and givinostat (1.44-fold change, p = 0.0076, two-way ANOVA, ASO versus ASO + givinostat), but not with EX527 (1.10-fold change, p = 0.7012, two-way ANOVA, ASO versus ASO + EX527) in both skeletal muscles and heart (Figure 2A). Dystrophin restoration and correct localization were also confirmed by immunostaining performed on muscle cryosections (Figure 2B). Compared with the treatment with ASO alone, we did not detect significantly more dystrophin-positive fibers in muscles from HDACi co-treated mice (approximately 3% of dystrophin-positive fibers in all groups), but the intensity of the staining was higher (Figure S2B), which fits with the higher amounts detected on western blot. Since some HDACi have previously been shown to increase muscle fiber size in *mdx* mice, we measured the muscle fiber sizes in the triceps of all groups of mice (Figures 2C and S2C). *Mdx* muscles display a higher number of small fibers (<1,000 μm^2^) compared with WT mice and ASO-treated mice, where the distribution shows a normalization toward WT muscles (fewer small fibers and more middle sized-fibers [2,000–4,000 μm^2^]). Co-treatment with givinostat tends to induce very large fibers (<5,000 μm^2^), as previously reported,^16^ and the mean fiber area was therefore significantly improved compared with *mdx* saline (Figures 2C and 2D). Previous studies have shown that the effects of HDACi on muscle histopathology are mediated by an upregulation of follistatin, an antagonist of both myostatin and activin A.^18^^,^^21^ We thus investigated the expression of follistatin in mice treated with ASO alone or in combination with HDACi and detected only a slight and not statistically significant elevation compared with the saline group (p = 0.2769) (Figure S2D). This suggests that the effect of HDACi on dystrophin expression and muscle fiber size are not or, at least, not completely due to increased follistatin expression.

To investigate whether the higher protein restoration obtained with the combined therapy ASO and HDACi could be due to higher mRNA levels or differences in transcript imbalance, we assessed *Dmd* gene expression at different exon junctions in co-treated mice as before. The qPCR results revealed higher levels of *Dmd* transcripts in the diaphragm (Figures 2E and S2E) of all treated groups of mice compared with saline *mdx* mice, but this was only statistically significant in *mdx* mice co-treated with VPA after multiple testing correction (p = 0.0001). In mice treated with HDACi alone (givinostat, VPA, or EX527), no statistical difference was detected (Figure S1B).

It has previously been demonstrated that two fragments of the myofibrillar structural protein myomesin-3 (MYOM3) are abnormally present in sera of the *mdx* mouse model and that this biomarker can be used to evaluate the efficacy of treatments.^9^^,^^22^ Levels of MYOM3 in the serum following treatment with ASO alone were significantly decreased by 57% (p < 0.0001) after 4 weeks of treatment (Figure 2F). MYOM3 levels were also significantly reduced with HDACi co-treatment (p < 0.0001 for all conditions compared with the saline group) although not statistically different from ASO alone.

### Effect of HDAC inhibitors on ASO biodistribution and toxicity

**Figure 3:**
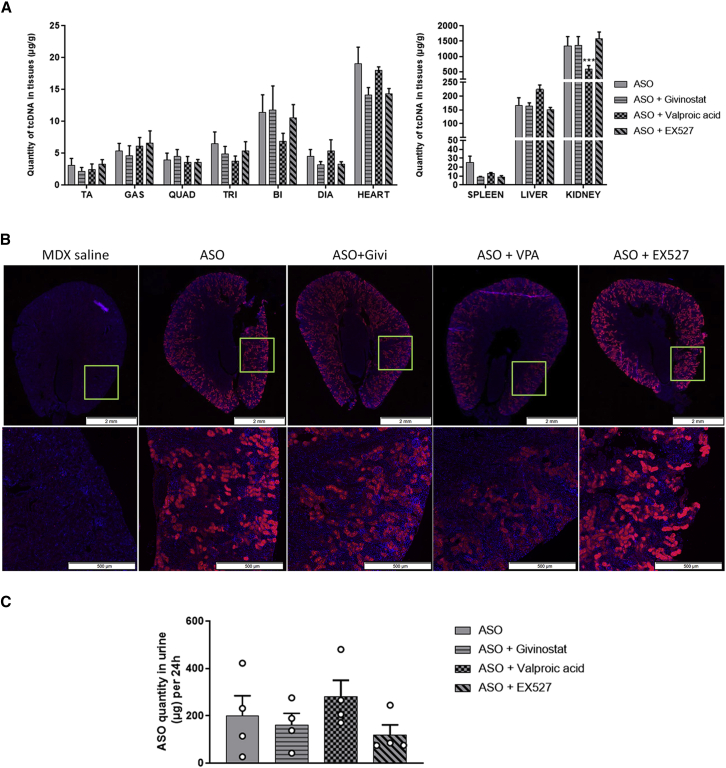
Impact of HDAC inhibitors on ASO biodistribution (A) Quantification of ASO in the different muscle tissues tibialis anterior (TA), gastrocnemius (GAS), quadriceps (QUAD), triceps (TRI), biceps (BI), diaphragm (DIA) and heart (left), and accumulation organs such as spleen, liver, and kidney (right) after 4 weeks of ASO treatment. n = 7 mice per group, ∗∗∗p < 0.001 compared with ASO analyzed by two-way ANOVA. (B) Detection of ASO (red staining) by *in situ* hybridization on transverse sections of kidneys from *mdx* mice treated with saline, ASO, or ASO + HDACi. Nuclei are labeled in blue (DAPI). Scale bars, 2,000 μm for the entire kidney sections (top panels) and 500 μm for the zoomed-in cortex regions (lower panels). The green squares indicate the location of the zoomed-in region. (C) ASO quantification in urine collected during 24 h after the last ASO injection (n = 4 mice per group). All data are plotted as means ± SEM.

To verify that increased skipping and protein restoration levels are not due to higher ASO concentrations in tissues, we checked the quantity of ASO in muscle tissues (Figure 3A). ASO quantification was performed as previously described^23^ and revealed similar levels of ASO in all muscle tissues (p = 0.5122, p = 0.4380, and p = 0.5257 for VPA, givinostat, and EX527, respectively). We calculated the ratio between protein restoration and ASO content, which reflects the therapeutic index of each treatment (Table 2). This highlights the superiority of the combined ASO + VPA therapy, since we found a nearly 2-fold increase between the two treatments (0.66 for ASO alone versus 1.26 for ASO + VPA). We also assessed the ASO content in liver, kidney, and spleen where ASOs tend to preferentially accumulate (Figure 3A, right). While we detected no difference between the different treatments in liver and spleen, we found a significant reduction (3-fold) in the kidneys of mice treated with ASO + VPA (p = 0.0009). This information is particularly promising for potential long-term treatment and safety, since the combined ASO + VPA therapy could lead to higher protein rescue in muscles with less ASO accumulation in kidney. Taking this result into account, we calculated the ratio between efficacy (average protein rescue across muscles) and ASO quantity in kidneys to determine the treatment with the best benefit/risk ratio. The combined therapy ASO + VPA revealed a near 4-fold improved ratio compared with ASO alone (Table 3).

**Table 2:** Ratio between protein rescue and ASO content in muscle tissues and fold change compared with treatment with ASO alone

Protein rescue/ASO quantification	TA	GAS	QUAD	TRI	DIA	HEART	Average	Fold change
ASO	0.93	0.86	0.82	0.49	1.33	0.22	0.66	1.00
ASO + GIVI	1.27	1.45	1.17	1.26	2.60	0.39	1.16	1.75
ASO + VPA	2.32	1.55	1.38	1.29	1.92	0.37	1.26	1.90
ASO + EX527	0.76	0.77	0.76	0.93	2.43	0.24	0.84	1.27

**Table 3:** Ratio between the average protein rescue across muscles and ASO content in kidneys, representing the benefit/risk of each combined therapy

	Average protein restoration (%)	ASO content in kidney (mg/g)	Benefit/risk ratio	Fold change compared with ASO
ASO	4.03	1.35	2.99	1.00
ASO + GIVI	5.80	1.37	4.24	1.42
ASO + VPA	6.99	0.59	11.81	3.95
ASO + EX527	4.44	1.58	2.81	0.94

The fold change is expressed in comparison with treatment with ASO alone.

We also checked ASO localization in the kidneys by *in situ* hybridization using a fluorescently labeled probe specific to the ASO. Following intravenous injections, the palm-tcDNA, like most charged ASOs, was found in the cortex area of kidneys and more specifically in proximal tubules^24^ (Figure 3B). Co-treatment with HDACi did not modify ASO localization, but the staining intensity was lower in VPA-treated mice, confirming the data obtained with the ASO quantification assay. Moreover, ASO quantification in urine revealed a higher amount of ASO in the urine of VPA-treated mice compared with mice treated with ASO alone (although not statistically significant), suggesting a tendency for better kidney clearance (Figure 3C).

**Figure 4:**
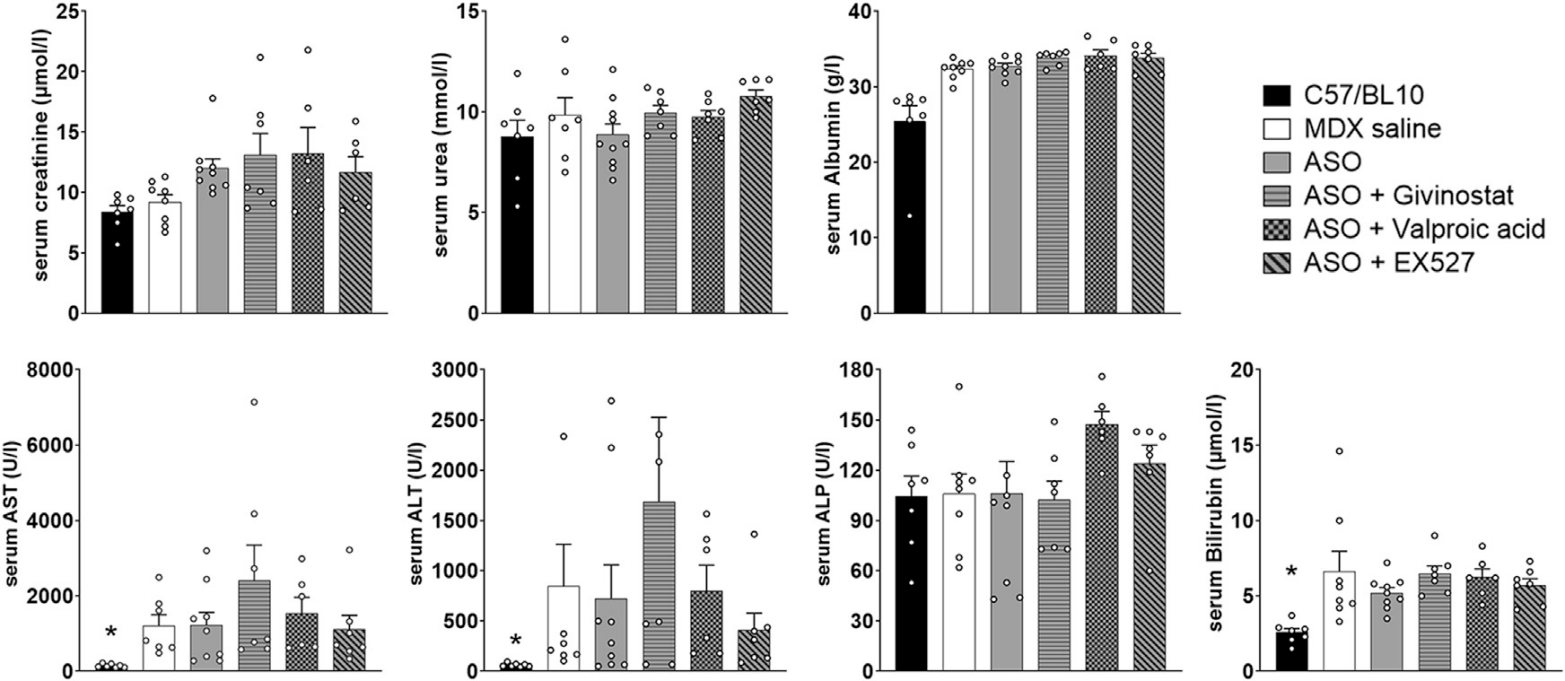
Serum biochemistry following HDACi treatment Quantification of general toxicity biomarkers in the serum: creatinine, urea, albumin, aspartate aminotransferase (AST), alanine aminotransferase (ALT), alkaline phosphatase (ALP), and bilirubin. n = 7 mice per group, ∗p < 0.05 compared with *mdx* saline, analyzed by Kruskal-Wallis test. All data are plotted as means ± SEM.

To ensure that the combination of HDACi with ASO did not induce any specific toxicity, we analyzed the serum levels of various general biomarkers in mice following the different treatments (Figure 4). Quantification of serum creatinine, urea, albumin, alkaline phosphatase (ALP), bilirubin, alanine aminotransferase (ALT), and aspartate aminotransferase (AST) revealed no significant changes in HDACi-treated *mdx* mice compared with saline-treated *mdx* mice. The slight elevation in AST and ALT levels observed in ASO + givinostat-treated mice was mostly due to a single individual and was not statistically significant, as was the effect of givinostat alone (Figure S3).

### Effect of valproic acid on chromatin organization

**Figure 5:**
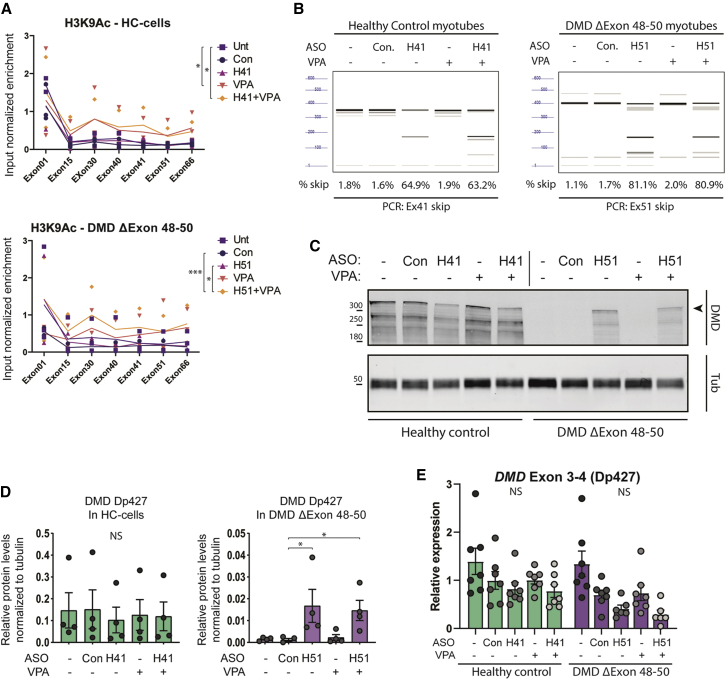
Effect of valproic acid on *DMD* chromatin organization and expression *in vitro* (A) ChIP-qPCR analysis of the histone mark H3K9Ac at various locations in the *DMD* locus in immortalized myocytes derived from a healthy control (HC) or a DMD patient. Cells were untreated (Unt), treated with a control ASO (targeting NOTCH3 [Con]), ASOs inducing exon skipping of *DMD* exon 41 (H41), *DMD* exon 51 (H51), valproic acid (VPA), or a combination of ASOs and VPA as indicated. Enrichment of H3K9Ac was normalized to input chromatin. n = 3 replicates, ∗p < 0.05, ∗∗∗p < 0.001, RM-ANOVA between two groups as indicated. (B) Quantification of exon skipping induced in a representative sample set corresponding to (A), as determined by analysis with Femto pulse. PCR around exon 41 in HC cells and PCR around exon 51 in DMD patient cells are shown; percentages below each lane indicate exon-skipping efficiency over the WT PCR product. Expected PCR product sizes for unskipped and skipped transcripts are 357 bp and 174 bp for exon 41 skipping, and 401 bp and 168 bp for exon 51, respectively. (C) Western blot analysis of DMD protein expression after combinatorial treatment with ASOs and VPA. HC or DMD immortal myocytes were treated with ASOs and/or VPA as indicated. Arrowhead indicates expected size of the DMD Dp427 protein. Tubulin was probed as a loading control for normalization purposes. (D) Quantification of n = 4 western blots, as presented in (C). Band intensity of DMD Dp427 was normalized to tubulin expression. Arbitrary units, n = 4 experiments, ∗p < 0.05, NS denotes non-significant; Kruskal-Wallis test. (E) qRT-PCR analysis of the expression of *DMD* Dp427 in myotube samples corresponding to the samples presented in (A) and (C) using isoform-specific primer sets. Gene expression was normalized to housekeeping genes *GUSB* and *GAPDH*. NS, not significant; Kruskal-Wallis test. All data are plotted as means ± SEM.

To further investigate the impact of VPA and the possible mechanisms underlying the higher dystrophin rescue induced by the combined ASO + VPA therapy, we next studied the effect of the combined treatment on *DMD* transcripts in cultured human myocytes. We first assessed whether changes in the chromatin landscape could be detected after treatment with ASOs and/or VPA. For this purpose, we treated immortalized myocytes derived from a healthy control (HC) or a DMD patient carrying a deletion of exons 48 through 50 with: a control ASO (not targeting DMD), an ASO targeting *DMD* exon 41 (generating an in-frame exon 41-skipped transcript in HC cells), or an ASO targeting *DMD* exon 51 (restoring the reading frame in these DMD cells); ASOs were tested alone as well as in combination with VPA treatment. As expected by blocking HDACs, treatment with VPA (alone and in combination with the ASO) increased the level of observed permissive histone mark H3K9Ac over the gene body of the *DMD* locus in HC cells, indicating that the *DMD* chromatin is indeed regulated through HDAC activity in the absence of pathogenic mutations (Figure 5A). No additional difference in H3K9Ac levels was observed for co-treatment of VPA with ASOs. Similarly, H3K9Ac levels were elevated in DMD patient cells treated with VPA, although the increase was only significant in DMD cells treated with ASO + VPA. Levels of the repressive histone mark H3K9me3 were not altered by treatment with VPA (Figure S4). None of the other histone marks that we investigated (H3K4me3, H3K27me3, and H3K36me3) showed any reproducible changes over the *DMD* locus caused by the treatment of cells with VPA and/or ASO (Figure S4).

Exon-skipping efficiency in the different *in vitro* samples was measured by RT-PCR followed by analysis of PCR product ratios using an Agilent Femto Pulse system (Figure 5B). Exon-skipping levels were comparable across VPA and ASO + VPA combination treatment for *DMD* exon 41 skipping in HC cells (∼64%) as well as for exon 51 skipping in DMD cells (∼81%), meaning that VPA did not induce changes in skipping efficiency in either cell type.

To assess whether VPA treatment affects production of dystrophin protein, we compared dystrophin recovery in samples treated with ASOs and ASO + VPA combination treatment using western blot that allows the detection of full-length dystrophin (Dp427) (Figure 5C). In HC cells, ASOs or ASO + VPA treatments had no significant impact on Dp427 expression (p > 0.9999) (Figure 5D). In DMD cells, ASO-mediated exon 51 skipping induces a distinct recovery of full-length dystrophin (Dp427) (p = 0.0407 and p = 0.0483 for ASO and ASO + VPA compared with control ASO, respectively), but no difference was observed between ASO alone or ASO + VPA (Figure 5D). The mRNA levels of Dp427 showed no significant alterations compared with the control ASO sample resulting from the described treatments (Figure 5E), although we observed a slight decrease (not statistically significant) in samples transfected with DMD-targeting ASOs (in both HC and DMD cells). Since DMD is primarily expressed in mature muscle cells, we looked at the expression levels of *MYOG* (Figure S5A) and *MYH3* (Figure S5B) as markers of myogenesis progression. We noted a loss of myogenic potential, as determined by *MYH3* levels in cells transfected with DMD-targeting ASOs (Figure S5B), which may explain the reduced levels of *DMD* transcripts in these samples.

Analysis of various exon-exon junctions of the *DMD* gene confirmed lower expression of *DMD* transcripts in DMD cells when compared with HC cells (Figure S5C), with a clear 5′-3′ imbalance, especially in the samples derived from the DMD patient (Figure S5C). The exon 3-4 junction seems equally abundant in HC and DMD cells, again suggesting that the ratio of transcription initiation is similar in either cell type, but the 5′-3′ transcript imbalance is clearer in DMD cells. However, we could not detect any significant changes in the imbalance following ASO or VPA treatment in DMD cells (Figure S5D).

## Discussion

In this study, we used three different HDACi: givinostat (a pan HDACi), VPA (inhibitor of HDAC class I and II), and EX527 (an inhibitor of sirtulin: HDAC class III) and combined them with an exon-skipping approach using tcDNA-based ASOs. We have previously demonstrated that palm-tcDNA ASOs represent promising drugs for the systemic treatment of DMD based on a 12-week treatment.^9^ In the present proof-of-concept study evaluating combined therapies, we deliberately used a short-term 4-week treatment, resulting in lower levels of dystrophin expression (approximately 3% of WT levels) in order to leave space for improvement.

The combined ASO + givinostat and ASO + VPA treatments induced significantly higher dystrophin restoration levels than ASO therapy alone (1.7- and 1.4-fold, respectively) as detected by western blot. Restoration of dystrophin was also confirmed by immunohistochemistry which did not reveal more dystrophin-positive fibers in muscles from HDACi co-treated mice, suggesting that the increase in dystrophin recovery is a consequence of higher intracellular levels of dystrophin rather than a higher number of cells expressing dystrophin. This would be in line with our hypothesis according to which HDACi treatment may increase available mRNA (leading to increased dystrophin production in each targeted nucleus) rather than have an impact on ASO biodistribution, which may have led to more dystrophin-positive fibers.

To investigate whether higher dystrophin restoration could have been due to unexpected higher uptake of ASO in muscle tissues, we checked ASO biodistribution after combined therapies. ASO amount in target muscle tissues was not altered by any of the HDACi, but VPA treatment surprisingly reduced the amount of ASO accumulated in kidneys. This may be due to higher kidney clearance given that more ASOs were found in the urine of VPA-treated mice, although this does not necessarily mean that the initial biodistribution to kidney was affected (perhaps only the clearance rate). While further work will be required to determine the underlying mechanisms, this represents an interesting advantage for the combined ASO + VPA therapy, which shows a 4-fold increase in the benefit/risk ratio.

HDACi have previously been studied in the context of DMD models but not directly for their effect on dystrophin transcript synthesis. Previous published work has shown how HDACs play a role in DMD pathophysiology and how they can mediate the connection between the dystrophin-associated glycoprotein complex (DAPC) and nuclear gene/microRNA (miRNA) expression. For example, expression of miRNA-1 in healthy muscle fibers occurs as a consequence of HDAC2 nitrosylation by nitric oxide synthase (NOS), which happens when NOS is bound to dystrophin at the DAPC. Nitrosylated HDAC2 is then released by the miRNA-1 locus with consequent gene expression.^25^ A similar connection has also been established for follistatin, which is controlled by class I HDACs, specifically HDAC2 (even though some evidence connecting follistatin expression with class II HDAC4 activity is also present). Inhibition of HDAC2 as well as reconstitution of the dystrophin-NOS signaling has been shown to result in derepression of follistatin. Pharmacological blockade of HDACs has been shown to decrease fibrosis and promote compensatory regeneration in the *mdx* skeletal muscle through this follistatin upregulation.^16^^,^^17^^,^^18^

In our study, we only detected a slight and not statistically significant upregulation of follistatin gene expression following HDACi treatment, suggesting that increased dystrophin expression was not directly linked. We further measured muscle fiber cross-sectional area (CSA) and demonstrated that givinostat indeed significantly increased fiber size area but not VPA. Givinostat treatment was previously shown to ameliorate morphology and muscular function in *mdx* mice by significantly reducing fibrosis in muscle tissue and promoting the increase of CSA of the muscles.^16^ It is possible that the increased level of dystrophin detected in ASO + givinostat-treated mice is due to the overall improvement of muscle histopathology and the increase in fiber size. However, CSA was not significantly impacted by the VPA treatment, suggesting a different underlying mechanism. When investigating whether combined therapies with HDACi had impacted the levels of dystrophin mRNA, we detected a significantly increased level of transcripts in VPA co-treated muscles. This may explain the higher dystrophin expression in ASO + VPA-treated mice and would be in line with our hypothesis which assumed that HDACi could increase the level of *Dmd* transcript and ultimately improve the efficacy of the exon-skipping approach.

While we observed a significant increase in VPA-induced dystrophin protein recovery *in vivo* in *mdx* mice treated with ASO + VPA, this effect was not recapitulated in the *in vitro* human myocyte model. This may be explained by the difference in treatment regimen between the two models, as the murine samples were treated with VPA for 5 weeks, as opposed to 24 h for human *in vitro* samples. Exon-skipping efficiency is also significantly higher in cultured cells than in muscles *in vivo*, likely due to the direct delivery of ASO to the cells. This high *in vitro* exon-skipping efficiency could potentially obscure subtle positive effects of VPA, which are observable *in vivo* where dystrophin recovery only reaches a fraction of WT levels. We also noted a loss of myogenic potential, as determined by *MYH3* levels in cells transfected with DMD-targeting ASOs, suggesting that the *in vitro* model may not be the most appropriate model to analyze subtle dystrophin expression changes.

HDACi have previously been shown to alter the myogenesis of various cultured myocytes models,^16^^,^^26^^,^^27^ but this was not readily observed in the ASO-treated cell lines treated with VPA in our study. However, most reports claiming increased myogenesis have used pan-HDAC inhibitors such as givinostat or trichostatin A.^16^^,^^27^ Given that VPA is an HDAC class I and IIa inhibitor, it may not inhibit the HDACs that cause the reported increase in myogenic potential.

Despite the lack of detectable increase in dystrophin levels, we observed increased H3K9Ac levels at the *DMD* gene upon treatment with VPA and combination of targeting ASO and VPA of human myocytes as assessed by chromatin immunoprecipitation followed by qPCR (ChIP-qPCR). This result confirms our hypothesis that predicted an impact of HDACi *DMD* transcript levels.

Of note, increased histone acetylation levels have been reported to cause spontaneous exon skipping in neuronal cells (in the neural cell adhesion molecule gene),^28^ presumably by the kinetic coupling of transcription and splicing.^29^ As such, it is possible that HDACi could facilitate ASO-mediated exon skipping (although this is not observed in our *in vitro* model). However, it is unclear as to what the threshold is for H3K9Ac to induce increased exon skipping, and whether our VPA-treated samples reach such levels.

Interestingly, Marasco et al. recently reported that co-treatment of VPA and ASOs could improve exon 7 inclusion in SMN2 transcripts.^30^ They showed that SMN2 exon 7 belongs to the class of type 2 exons where acetylation of H3K9 improves RNA polymerase II speed and inclusion and H3K9me2 results in slower RNA polymerase II and exon 7 skipping. They also observed that ASO treatment resulted in increased dimethylation in SMN2 intron 7. Thus, while the net effect of ASO treatment was more exon 7 inclusion, the dimethylation decreased exon 7 inclusion. Co-treatment with VPA reduced dimethylation and increased ASO-induced exon 7 inclusion. We did not assess local chromatin changes in the targeted regions. Furthermore, we target exonic regions in our study while Marasco et al. targeted an intronic region. In either case, we did not observe a clear effect of VPA treatment on exon-skipping levels but rather on dystrophin protein levels. This suggests that an effect like the one described for SMN2 intron 7 did not occur and that our positive effects are rather due to increased expression of dystrophin transcripts.

It has recently been reported that HDACi have an influence on the activity of other cell types present in the muscle tissue, such as fibro-adipogenic progenitors and muscle stem cells.^31^ The altered microenvironment influenced by HDACi might improve general muscle tissue health and, concomitantly, result in improved delivery of the ASO and/or recovery of ASO-induced dystrophin protein. This potential positive effect of HDACi + VPA is not properly mimicked in the homogeneous *in vitro* myocyte cultures, potentially explaining why this model does not benefit from HDACi in similar fashion to our *in vivo* model system.

In conclusion, our results show a clear beneficial effect of VPA on the recovery of dystrophin protein in *mdx* mice after exon skipping and confirm that even short treatments with VPA have a noticeable effect on the chromatin marks present at the *DMD* locus. However, whether these two observations are causative or correlative is currently unclear and warrants further research into the epigenetic status of the *Dmd* locus in muscle tissue of VPA-treated *mdx* mice. Moreover, it would be useful to evaluate the functional outcomes of this increased dystrophin expression in future studies.

Considering that the HDACi used in this study have either been approved in the clinic (VPA) or are being evaluated in late-stage clinical trials (givinostat), this work provides encouraging data supporting a future combined therapy which could be rapidly translated to the clinic.

## Materials and methods

### Antisense oligonucleotides and animal experiments

Animal procedures were performed in accordance with national and European legislation, approved by the French government (Ministère de l’Enseignement Supérieur et de la Recherche, Autorisation APAFiS #6518). *Mdx* (C57BL/10ScSc-Dmdmdx/J) mice were bred in our animal facility at the Plateforme 2Care, UFR des Sciences de la Santé, Université de Versailles Saint Quentin, and were maintained in a standard 12:12-h light/dark cycle with free access to food and water. Mice were weaned at weeks 4–5 postnatal, and 2–5 individuals were housed per cage.

tcDNA-ASO targeting the donor splice site of exon 23 of the mouse dystrophin pre-mRNA^32^ were synthesized by SQY Therapeutics (Montigny le Bretonneux, France). Palmitic acid was conjugated at the 5′ end of tcDNA-PO via a C6-amino linker and a phosphorothioate bond as previously described.^9^

Groups of 8- to 10-week-old *mdx* mice were treated with HDAC inhibitors or saline (n = 7 mice per group) for 5 weeks as follows: VPA (Santa Cruz, dissolved in PBS and used at a final concentration of 500 mg/kg/day) and EX527, also known as Selisistat or SEN0014196 (ADOOQ Bioscience, dissolved in PBS 2% dimethyl sulfoxide [DMSO] and used at a final concentration of 2 mg/kg/day) were injected i.p. five times per week as previously described;^19^^,^^20^ givinostat (ADOOQ Bioscience. dissolved in H_2_O 2% DMSO and used at a final concentration of 10 mg/kg/day) was administered by oral gavage as previously described.^13^ After the first week of HDACi treatment, mice were treated with ASO (one intravenous injection per week at 25 mg/kg/week under general anesthesia using 2% isoflurane). Age-matched *mdx* groups receiving an equivalent volume of sterile saline were included as controls, and C57BL/10 mice were included as WT controls.

Animals were euthanized 2 weeks after the last ASO injection; thereafter, muscles and tissues were harvested and snap-frozen in liquid-nitrogen-cooled isopentane and stored at −80°C before further analysis. To assess the biodistribution of the ASO treatment, kidneys were sampled at the end of the protocol, fixed in 10% neutral buffered formalin, and embedded in paraffin wax. Blood samples were also collected at the end of the treatment for MYOM-3 and biochemistry analysis. Analyses of serum ALT, AST, ALP, bilirubin, creatinine, urea, and albumin levels were performed by the pathology laboratory at the Mary Lyon Centre, Medical Research Council, Harwell, Oxfordshire, UK.

### ASO quantification by fluorescent hybridization assay

Tissues were homogenized using the Precellys 24 (Bertin Instruments, France) in lysis buffer (100 mmol/L Tris-HCl [pH 8.5], 200 mmol/L NaCl, 5 mmol/L EDTA, 0.2% SDS) containing 2 mg/mL of proteinase K (Invitrogen) (50 mg tissue/mL of buffer), followed by incubation overnight at 55°C in a hybridization oven. After centrifugation at 7,000 × *g* (Sorval ST 8R centrifuge, 75005719 rotor) for 15 min, the supernatant was used in the assay. Quantification of ASO was performed using a hybridization assay with a molecular beacon probe, as previously described.^23^ In brief, 10 μL of tissue lysates or serum was incubated with a 5′ Cy3-DNA complementary probe conjugated with HBQ quencher at 3′ in black non-binding 96-well plates (Thermo Fisher Scientific, USA). PBS was added to a final volume of 100 μL per well, and fluorescence was measured on a spectrophotometer (excitation 544 nm/emission 590 nm using FluoStar Omega). The amount of tcDNA in tissues was determined using a standard curve built on the measurement of known tcDNA quantities dissolved in the respective tissue lysates of mock-injected animals.

### RNA analysis

Total RNA was isolated from snap-frozen muscle tissues using TRIzol reagent according to the manufacturer’s instructions (Thermo Fisher Scientific).

To visualize exon-skipping levels, aliquots of 500 ng of total RNA were used for RT-PCR analysis using the Access RT-PCR System (Promega, USA) in a 25-μL reaction using the external primers Ex 20Fo (5′-CAG AAT TCT GCC AAT TGC TGA G-3′) and Ex 26Ro (5′-TTC TTC AGC TTG TGT CAT CC-3′). The cDNA synthesis was carried out at 45°C for 45 min, directly followed by the primary PCR of 29 cycles of 95°C (30 s), 55°C (1 min), and 72°C (2 min). 1.5 μL of these reactions was then reamplified in nested PCRs by 24 cycles of 95°C (30 s), 55°C (1 min), and 72°C (2 min) using the internal primers Ex 20Fi (5′-CCC AGT CTA CCA CCC TAT CAG AGC-3′) and Ex 26Ri (5′-CCT GCC TTT AAG GCT TCC TT-3′). PCR products were analyzed on 1.5% agarose gels with ethidium bromide.

Exon 23 skipping levels were quantified using real-time qPCR using TaqMan assays designed against the exon 23–24 junction and exon 22–24 junction as previously described.^9^ Seventy nanograms of cDNA was used as input per reaction, and all assays were carried out in triplicate. Assays were performed under fast cycling conditions on a Bio-Rad CFX384 Touch Real-Time PCR Detection System, and all data were analyzed using the absolute copy number method. For a given sample the copy number of skipped product (exon 22–24 assay) and unskipped product (exon 23–24 assay) were determined using the standards Ex20-26 and Ex20-26Delta23 respectively (gBlocks gene fragments from Integrated DNA Technologies). Exon 23 skipping was then expressed as a percentage of total dystrophin (calculated by the addition of exon 22–23 and exon 22–24 copy numbers). Quantification of Dmd transcripts at different exon-exon junctions was performed similarly, with probes targeting ex4-5 junction (Mm.PT.58.41636025), ex49-50 junction (Mm.PT.58.6233636), ex58-59 junction (Mm.PT.58.43613256), ex65-66 junction (Mm.PT.58.42993407), and GAPDH (Mm.PT.39a.1) (Integrated DNA Technologies). Absolute quantification was determined for each probe using the corresponding standards (gBlocks gene fragments from Integrated DNA Technologies).

Exon-skipping levels of samples derived from *in vitro* cultures were determined using RT-PCR with exon-specific primers (Table S1) followed by analysis of skipping ratios using an Agilent Femto Pulse. Peaks were called, and molarity of each PCR product was determined using the corresponding ProSize data analysis software (Agilent). Further data analysis and visualization was performed using GraphPad Prism version 9.

Follistatin quantification was performed using real-time qPCR using TaqMan assays (Mm.PT.58.11399784 from IDT). ΔΔCt analysis was performed with GAPDH as normalization.

### Western blot analysis

Protein lysates were obtained from intervening muscle sections collected during cryosection using the Precellys 24 (Bertin Instruments) in RIPA buffer (Thermo Fisher Scientific) complemented with SDS powder (5% final) (Bio-Rad, France) and protease inhibitor cocktail (Thermo Fisher Scientific). Protein extracts were denatured at 100°C for 3 min and centrifuged at 13,000 rpm for 10 min at 10°C. Supernatants were collected, and the total protein concentration was determined with the BCA Protein Assay Kit (Thermo Fisher Scientific). Twenty-five micrograms of protein was loaded onto NuPAGE 3%–8% Tris-acetate protein gels (Invitrogen), following the manufacturer’s instructions. Dystrophin protein was stained using iBind Flax Western Device (Thermo Fisher Scientific), and the membrane was labeled with NCL-DYS1 primary monoclonal antibody (NCL-DYS1, dilution 1:200; Novocastra, Newcastle, UK) and hVin-1 primary antibody (dilution 1:4,000; Sigma), followed by incubation with a goat anti-mouse secondary antibody (IRDye 800CW Goat anti-mouse immunoglobulin G [IgG], dilution 1:2,000; Li-Cor, Germany). Bands were visualized using the Odyssey CLx system (Li-Cor), and quantification was carried out using the Empiria Studio software (Li-Cor) based on a standard curve made from pooled lysates from C57BL10 (WT) and *mdx* control for each tissue.

For myomesin-3 detection, mouse sera were diluted at 1:20 before loading onto 3%–8% Criterion XT Tris-Acetate Protein Gel, following the manufacturer’s instructions (Bio-Rad). Myomesin-3 protein was detected by probing the nitrocellulose membrane with MYOM3 primary rabbit polyclonal antibody (MYOM3; Proteintech, Manchester, UK), followed by incubation with a goat anti-rabbit secondary antibody (IRDye 800CW Goat anti-rabbit IgG; Li-Cor). Bands were visualized using the Odyssey Imaging System (Biosciences, Lincoln, NE, USA). Signal intensities in treated samples were quantified and normalized to PBS control mouse signals using Image Studio software (Li-Cor).

For protein samples derived from i*n vitro* cell cultures, cells were lysed in 1× Laemmli sample buffer (2% SDS, 10% glycerol, 60 mM Tris [pH 6.8]) and denatured at 95°C. After normalization of protein concentration determined by the BCA Protein Assay Kit, samples were supplemented with 2% β-mercaptoethanol and 0.01% bromophenol blue prior to PAGE. The Immobulon-FL PVDF membrane (Merck-Millipore) was probed for dystrophin using ab154168 (Abcam) and tubulin T6199 (Sigma-Aldrich) in Takara immunobooster (Takara), followed by secondary antibodies donkey-α-Rabbit IRDye-800CW and donkey-α-Mouse IRDye-680RD (926-68072). The membrane was visualized using an Odyssey CLx infrared imaging system (Li-Cor) and analyzed using the accompanying Image Studio software v5.2 (Li-Cor).

### Immunohistochemistry analysis

Sections of 10 μm were cut from triceps and heart and examined for dystrophin expression using the rabbit polyclonal antibody dystrophin (dilution 1:500; Thermo Fisher Scientific, cat. no. RB-9024-P), which was then detected by donkey anti-rabbit IgG Alexa 594 (dilution 1:400; Jackson ImmunoResearch). Controls prepared by omitting primary antibody showed no specific staining. Images were taken at equivalent locations and exposure times using a Zeiss Axio Imager with an Orkan camera (Hamamatsu) and AxioVision software, and analyzed with ImageJ software.

To visualize ASO in kidney, 5-μm sections were cut from paraffin blocks, dewaxed (3× 5 min in xylene and 3× 3 min in ethanol 100) and stained with a complementary DNA probe fluorescently labeled with Alexa 594. Controls prepared by omitting the probe showed no specific staining. Scans were taken at equivalent exposure times using an IX83 inverted microscope (Olympus) with an Orca-R2 camera (Hamamatsu) and cellSens Dimension software.

For measurement of muscle fiber size, sections of 10 μm were cut from triceps and stained with the rabbit polyclonal antibody laminin (dilution 1:200; Sigma, ref. L9393-2ML), which was then detected by donkey anti-rabbit IgG Alexa 488 (dilution 1:400; Thermo Fisher Scientific, ref. A11008). Images were taken using a Zeiss Axio Imager with an Orkan camera (Hamamatsu) and AxioVision software, and analyzed with ImageJ software.

### Cell culture

Immortalized HC and DMD patient cell lines were kindly provided by Prof. Vincent Mouly (Institute of Myology, Paris).^33^^,^^34^ Cells were cultured in skeletal muscle growth medium (SMGM; Promokine), supplemented with 15% heat-inactivated fetal bovine serum (HI-FBS) and gentamycin. To induce differentiation of proliferating myoblasts to myotubes, SMGM medium was replaced with fusion medium consisting of Dulbecco’s modified Eagle’s medium (DMEM)-Glutamax, high-glucose (Gibco), supplemented with 2% HI-FBS and 1% penicillin-streptomycin (Gibco). Cells were maintained at 37°C and 5% CO_2_ in humidified incubators and were routinely tested for mycoplasma infection by the use of the Mycoalert Kit (Lonza). For transfection of myocytes with ASO, a 200-nM end concentration of ASO was transfected at day 3 of myogenic differentiation for 3 h, after which cells were left to further differentiate for 48 h as indicated. In brief, 200 nM end concentration of ASO (2′ O-methyl RNA; phosphorothioate backbone) (H41, cuccucuuucuucuucugc; H51, ucaaggaagauggcauuucu; Control-NOTCH3 (h4c12), agcagaggaagcguccauc, which is very inefficient at skipping NOTCH3 exon 4 by itself and known to not induce motif-related events such as an inflammatory response^35^) was mixed with a 1:4 ratio of Lipofectamine 2000 (Thermo Fisher Scientific), incubated at room temperature for 20 min, and added to culture dishes. After 3 h of incubation, transfection medium was replaced with fusion medium for further differentiation of myocytes. For VPA treatment, fusion medium was supplemented with 3 mM VPA (Sigma-Aldrich, ref. P4543-25G) freshly dissolved in Milli-Q® water for 24 h before harvesting as indicated.

### Chromatin immunoprecipitation

ChIP-qPCR was performed as previously described.^36^ In brief, after described treatments cells were crosslinked for 10 min by addition of formaldehyde to 1% final concentration. Fixation was quenched by addition of 125 mM glycine, after which cells were washed in cold PBS and collected. Nuclei were liberated from cells by resuspension in NP-ChIP buffer (150 mM NaCl, 5 mm EDTA, 0.5% Igepal CA-630, 1% Triton X-100 in 50 mM Tris-HCl [pH 7.5]), which were sheared for 25 cycles (30 s on, 30 s off) using a Bioruptor Pico (Diagenode). For each histone mark described, 3 μg of chromatin was precleared and incubated with antibodies (Table S2) overnight. Chromatin-antibody immunocomplexes were captured for 2 h using 20 μL of a mix of protein A and protein G Dynabeads (Life Technologies) at a 3:1 ratio. Beads were washed in a series of wash buffers: low salt buffer (0.1% SDS, 1% Triton X-100, 2 mM EDTA, 150 mM NaCl in 20 mM Tris-HCl [pH 8]), high salt buffer (0.1% SDS, 1% Triton X-100, 2 mM EDTA, 300 mM NaCl in 20 mM Tris-HCl [pH 8]), lithium chloride wash buffer (0.25 M LiCl, 1% Igepal CA-630, 1% sodium deoxycholate, 1 mM EDTA in 10 mM Tris-HCl [pH 8]), and twice with TE-wash buffer (1 mM EDTA in 10 mM Tris-HCl [pH 8]). DNA was eluted from the beads by boiling at 95°C for 10 min in the presence of 10% Chelex-100 resin (Bio-Rad). Corresponding input samples were phenol-chloroform isolated from 10% of input material used for each chromatin sample. Samples were analyzed using locus-specific primer sets (Table S1) and SensiMix Sybr HI-ROX (Bioline), run in a CFX-384 Real-Time PCR system (Bio-Rad). Values from histone ChIP samples were normalized to corresponding input samples using ΔCt calculations.

### Statistical analysis

All *in vivo* data were analyzed with GraphPad Prism8 software (GraphPad, San Diego, CA, USA) and expressed as means ± SEM. “n” refers to the number of mice per group.

Group comparisons were performed using one- and two-way ANOVA with repeated-measures comparisons when needed (e.g., effects at different exon junctions or in different muscle tissues), followed by post hoc Dunnett’s or Sidak’s multiple comparisons when appropriate. To compare the overall effect of two treatments across the different tissues or different exon junctions, the two groups were directly compared using a two-way ANOVA, and the p value of the treatment effect is indicated in the figure legends. The Kruskal-Wallis test was used to compare groups that did not follow a normal distribution (assessed with the Shapiro-Wilk test).

The statistical analysis of gene expression and transcript imbalance assessed by qPCR at various exon-exon junctions was also performed using a linear model, in which exons were included as a covariate (continuous) and group was included as a factor. Pairwise contrasts were assessed using the *emmeans* R package, and multiple testing correction was performed with the Tukey method.

Statistical analysis of all *in vitro* data was performed using GraphPad Prism version 9. Kruskal-Wallis test followed by Dunn’s multiple correction was performed as indicated. Repeated-measures (RM)-ANOVA was used as indicated. Data are plotted as means ± SEM. “n” refers to independently acquired and processed samples (biological replicates) as indicated. Biological replicates consist of technical triplicates in the case of qRT-PCR and ChIP-qPCR measurements. Significance levels were set at ∗p < 0.05, ∗∗p < 0.01, ∗∗∗p < 0.001, and ∗∗∗∗p < 0.0001.

## Data Availability

The primary data for this study are available from the authors upon request.

## Supplemental information

Document S1. Figures S1–S5 and Tables S1 and S2

Document S2. Article plus supplemental information

## References

[1] Fortunato F., Rossi R., Falzarano M.S., Ferlini A. (2021). Innovative therapeutic approaches for duchenne muscular dystrophy. J. Clin. Med..

[2] Clemens P.R., Rao V.K., Connolly A.M., Harper A.D., Mah J.K., Smith E.C., McDonald C.M., Zaidman C.M., Morgenroth L.P., Osaki H. (2020). Safety, tolerability, and efficacy of viltolarsen in boys with duchenne muscular dystrophy amenable to exon 53 skipping: a phase 2 randomized clinical trial. JAMA Neurol..

[3] Mitelman O., Abdel-Hamid H.Z., Byrne B.J., Connolly A.M., Heydemann P., Proud C., Shieh P.B., Wagner K.R., Dugar A., Santra S. (2022). A combined prospective and retrospective comparison of long-term functional outcomes suggests delayed loss of ambulation and pulmonary decline with long-term eteplirsen treatment. J. Neuromuscul. Dis..

[4] Godfrey C., Desviat L.R., Smedsrød B., Piétri-Rouxel F., Denti M.A., Disterer P., Lorain S., Nogales-Gadea G., Sardone V., Anwar R. (2017). Delivery is key: lessons learnt from developing splice-switching antisense therapies. EMBO Mol. Med..

[5] Hammond S.M., Aartsma-Rus A., Alves S., Borgos S.E., Buijsen R.A.M., Collin R.W.J., Covello G., Denti M.A., Desviat L.R., Echevarría L. (2021). Delivery of oligonucleotide-based therapeutics: challenges and opportunities. EMBO Mol. Med..

[6] Ferlini A., Goyenvalle A., Muntoni F. (2021). RNA-targeted drugs for neuromuscular diseases. Science.

[7] Roberts T.C., Langer R., Wood M.J.A. (2020). Advances in oligonucleotide drug delivery. Nat. Rev. Drug Discov..

[8] Hammond S.M., Hazell G., Shabanpoor F., Saleh A.F., Bowerman M., Sleigh J.N., Meijboom K.E., Zhou H., Muntoni F., Talbot K. (2016). Systemic peptide-mediated oligonucleotide therapy improves long-term survival in spinal muscular atrophy. Proc. Natl. Acad. Sci. USA.

[9] Relizani K., Echevarría L., Zarrouki F., Gastaldi C., Dambrune C., Aupy P., Haeberli A., Komisarski M., Tensorer T., Larcher T. (2022). Palmitic acid conjugation enhances potency of tricyclo-DNA splice switching oligonucleotides. Nucleic Acids Res..

[10] Chamberlain J.S., Pearlman J.A., Muzny D.M., Gibbs R.A., Ranier J.E., Caskey C.T., Reeves A.A. (1988). Expression of the murine Duchenne muscular dystrophy gene in muscle and brain. Science.

[11] Haslett J.N., Sanoudou D., Kho A.T., Bennett R.R., Greenberg S.A., Kohane I.S., Beggs A.H., Kunkel L.M. (2002). Gene expression comparison of biopsies from Duchenne muscular dystrophy (DMD) and normal skeletal muscle. Proc. Natl. Acad. Sci. USA.

[12] Gehring N.H., Kunz J.B., Neu-Yilik G., Breit S., Viegas M.H., Hentze M.W., Kulozik A.E. (2005). Exon-junction complex components specify distinct routes of nonsense-mediated mRNA decay with differential cofactor requirements. Mol. Cell.

[13] García-Rodríguez R., Hiller M., Jiménez-Gracia L., van der Pal Z., Balog J., Adamzek K., Aartsma-Rus A., Spitali P. (2020). Premature termination codons in the DMD gene cause reduced local mRNA synthesis. Proc. Natl. Acad. Sci. USA.

[14] Spitali P., van den Bergen J.C., Verhaart I.E.C., Wokke B., Janson A.A.M., van den Eijnde R., den Dunnen J.T., Laros J.F.J., Verschuuren J.J.G.M., ’t Hoen P.A.C., Aartsma-Rus A. (2013). DMD transcript imbalance determines dystrophin levels. FASEB J.

[15] Tennyson C.N., Shi Q., Worton R.G. (1996). Stability of the human dystrophin transcript in muscle. Nucleic Acids Res..

[16] Consalvi S., Mozzetta C., Bettica P., Germani M., Fiorentini F., Del Bene F., Rocchetti M., Leoni F., Monzani V., Mascagni P. (2013). Preclinical studies in the mdx mouse model of duchenne muscular dystrophy with the histone deacetylase inhibitor givinostat. Mol. Med..

[17] Licandro S.A., Crippa L., Pomarico R., Perego R., Fossati G., Leoni F., Steinkühler C. (2021). The pan HDAC inhibitor Givinostat improves muscle function and histological parameters in two Duchenne muscular dystrophy murine models expressing different haplotypes of the LTBP4 gene. Skelet. Muscle.

[18] Minetti G.C., Colussi C., Adami R., Serra C., Mozzetta C., Parente V., Fortuni S., Straino S., Sampaolesi M., Di Padova M. (2006). Functional and morphological recovery of dystrophic muscles in mice treated with deacetylase inhibitors. Nat. Med..

[19] Gurpur P.B., Liu J., Burkin D.J., Kaufman S.J. (2009). Valproic acid activates the PI3K/Akt/mTOR pathway in muscle and ameliorates pathology in a mouse model of Duchenne muscular dystrophy. Am. J. Pathol..

[20] Daenthanasanmak A., Iamsawat S., Chakraborty P., Nguyen H.D., Bastian D., Liu C., Mehrotra S., Yu X.-Z. (2019). Targeting Sirt-1 controls GVHD by inhibiting T-cell allo-response and promoting Treg stability in mice. Blood.

[21] Iskenderian A., Liu N., Deng Q., Huang Y., Shen C., Palmieri K., Crooker R., Lundberg D., Kastrapeli N., Pescatore B. (2018). Myostatin and activin blockade by engineered follistatin results in hypertrophy and improves dystrophic pathology in mdx mouse more than myostatin blockade alone. Skelet. Muscle.

[22] Rouillon J., Poupiot J., Zocevic A., Amor F., Léger T., Garcia C., Camadro J.M., Wong B., Pinilla R., Cosette J. (2015). Serum proteomic profiling reveals fragments of MYOM3 as potential biomarkers for monitoring the outcome of therapeutic interventions in muscular dystrophies. Hum. Mol. Genet..

[23] Echevarría L., Aupy P., Relizani K., Bestetti T., Griffith G., Blandel F., Komisarski M., Haeberli A., Svinartchouk F., Garcia L., Goyenvalle A. (2019). Evaluating the impact of variable phosphorothioate content in tricyclo-DNA antisense oligonucleotides in a duchenne muscular dystrophy mouse model. Nucleic Acid Ther..

[24] Hung G., Xiao X., Peralta R., Bhattacharjee G., Murray S., Norris D., Guo S., Monia B.P. (2013). Characterization of target mRNA reduction through in situ RNA hybridization in multiple organ systems following systemic antisense treatment in animals. Nucleic Acid Ther..

[25] Cacchiarelli D., Martone J., Girardi E., Cesana M., Incitti T., Morlando M., Nicoletti C., Santini T., Sthandier O., Barberi L. (2010). MicroRNAs involved in molecular circuitries relevant for the Duchenne muscular dystrophy pathogenesis are controlled by the dystrophin/nNOS pathway. Cell Metab..

[26] Breuls N., Giarratana N., Yedigaryan L., Garrido G.M., Carai P., Heymans S., Ranga A., Deroose C., Sampaolesi M. (2021). Valproic acid stimulates myogenesis in pluripotent stem cell-derived mesodermal progenitors in a NOTCH-dependent manner. Cell Death Dis..

[27] Iezzi S., Di Padova M., Serra C., Caretti G., Simone C., Maklan E., Minetti G., Zhao P., Hoffman E.P., Puri P.L., Sartorelli V. (2004). Deacetylase inhibitors increase muscle cell size by promoting myoblast recruitment and fusion through induction of follistatin. Dev. Cell.

[28] Schor I.E., Rascovan N., Pelisch F., Alló M., Kornblihtt A.R. (2009). Neuronal cell depolarization induces intragenic chromatin modifications affecting NCAM alternative splicing. Proc. Natl. Acad. Sci. USA.

[29] Saldi T., Cortazar M.A., Sheridan R.M., Bentley D.L. (2016). Coupling of RNA polymerase II transcription elongation with pre-mRNA splicing. J. Mol. Biol..

[30] Marasco L.E., Dujardin G., Sousa-Luís R., Liu Y.H., Stigliano J.N., Nomakuchi T., Proudfoot N.J., Krainer A.R., Kornblihtt A.R. (2022). Counteracting chromatin effects of a splicing-correcting antisense oligonucleotide improves its therapeutic efficacy in spinal muscular atrophy. Cell.

[31] Consalvi S., Tucciarone L., Macrì E., De Bardi M., Picozza M., Salvatori I., Renzini A., Valente S., Mai A., Moresi V., Puri P.L. (2022). Determinants of epigenetic resistance to HDAC inhibitors in dystrophic fibro-adipogenic progenitors. EMBO Rep..

[32] Relizani K., Griffith G., Echevarría L., Zarrouki F., Facchinetti P., Vaillend C., Leumann C., Garcia L., Goyenvalle A. (2017). Efficacy and safety profile of tricyclo-DNA antisense oligonucleotides in duchenne muscular dystrophy mouse model. Mol. Ther. Nucleic Acids.

[33] Echigoya Y., Lim K.R.Q., Trieu N., Bao B., Miskew Nichols B., Vila M.C., Novak J.S., Hara Y., Lee J., Touznik A. (2017). Quantitative antisense screening and optimization for exon 51 skipping in duchenne muscular dystrophy. Mol. Ther..

[34] Mamchaoui K., Trollet C., Bigot A., Negroni E., Chaouch S., Wolff A., Kandalla P.K., Marie S., Di Santo J., St Guily J.L. (2011). Immortalized pathological human myoblasts: towards a universal tool for the study of neuromuscular disorders. Skelet. Muscle.

[35] Rutten J.W., Dauwerse H.G., Peters D.J.M., Goldfarb A., Venselaar H., Haffner C., van Ommen G.-J.B., Aartsma-Rus A.M., Lesnik Oberstein S.A.J. (2016). Therapeutic NOTCH3 cysteine correction in CADASIL using exon skipping: in vitro proof of concept. Brain.

[36] Nelson J.D., Denisenko O., Bomsztyk K. (2006). Protocol for the fast chromatin immunoprecipitation (ChIP) method. Nat. Protoc..

