# Supplementary material for: Histone deacetylase inhibitors improve antisense-mediated exon-skipping efficacy in mdx mice
Source: Mol Ther Nucleic Acids. 2022 Nov 21;30:606–20. doi: 10.1016/j.omtn.2022.11.017 (PMC9722397; doi:10.1016/j.omtn.2022.11.017)
Supplement: Document S1. Figures S1–S5 and Tables S1 and S2 [file mmc1.pdf]

OMTN, Volume *30*

Supplemental information

Histone deacetylase inhibitors improve antisense-mediated exon-skipping efficacy in *mdx* mice

Flavien Bizot, Remko Goossens, Thomas Tensorer, Sergei Dmitriev, Luis Garcia, Annemieke Aartsma-Rus, Pietro Spitali, and Aurélie Goyenvalle

Supplemental Information

A

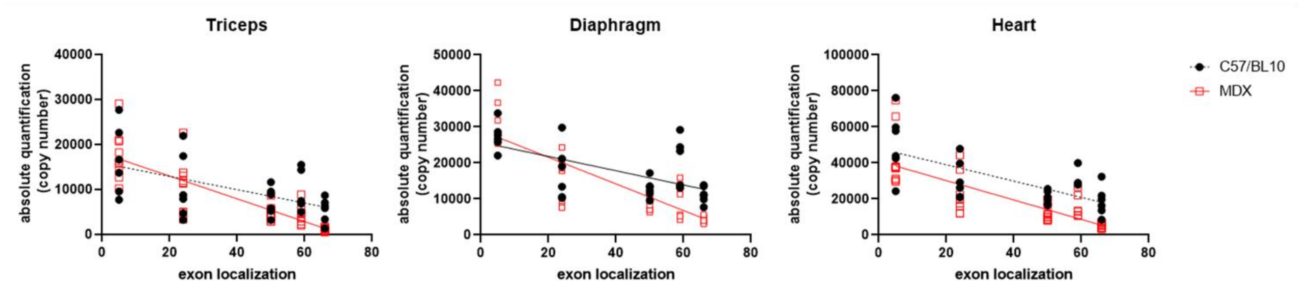

Slope:
C57/BL10: -148.2
MDX: -252.9

Slope:
C57/BL10: -198.3
MDX: -370.1

Slope:
C57/BL10: -453.6
MDX: -539.3

B

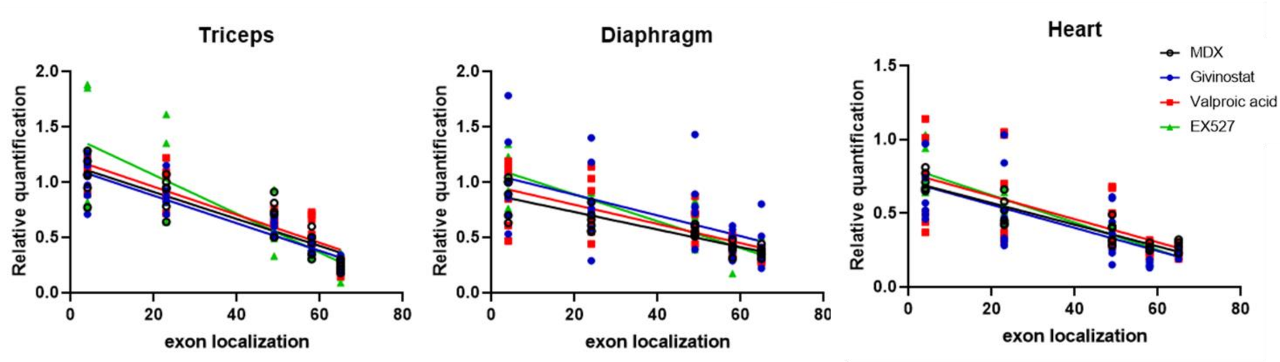

Figure S1: *Dmd* transcript levels in *mdx* and wt mice

(A) Absolute quantification of *Dmd* transcript levels obtained in triceps, diaphragm and heart for several exon–exon junctions along the *Dmd* gene in *mdx* and C57Bl10 mice (N=6 for C57Bl10 and 9 for *mdx* mice), normalized to GAPDH expression. (B) Relative quantification of *Dmd* transcript levels obtained in triceps, diaphragm and heart for several exon–exon junctions along the *Dmd* gene (N=6 mice per group), normalized to C57Bl10 mice.

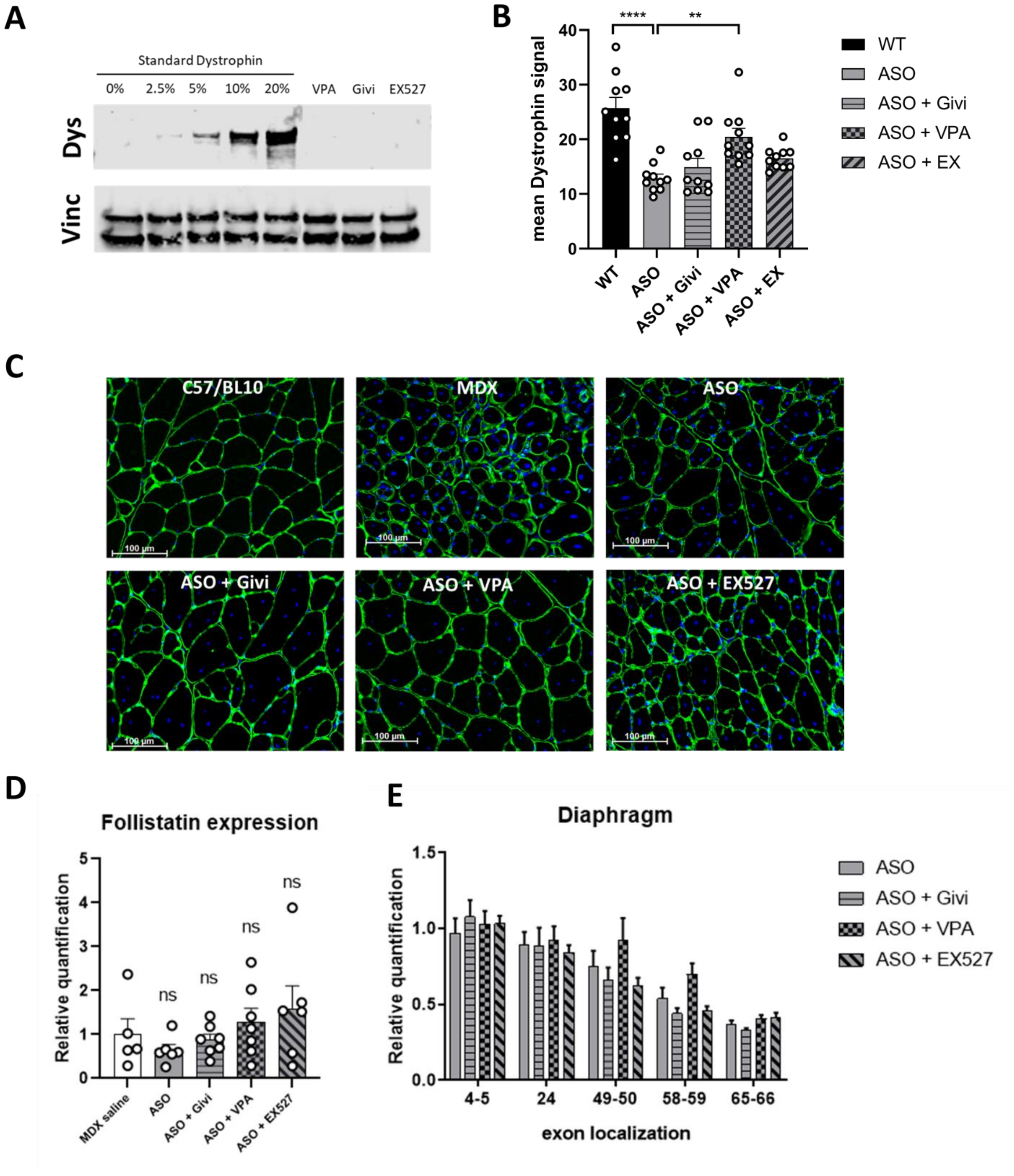

Figure S2: Histological and molecular characterization of muscles treated with ASO+HDACi.

(A) Western blot showing no dystrophin restoration in triceps from *mdx* mice treated with HDACi only. Vinculin is used for normalization (bottom panel). A standard curve made from pooled lysates from C57BL10 (WT) and *mdx* control was loaded as control (0%, 2.5%, 5%, 10% and 20% of WT). (B) Quantification of the dystrophin staining intensity in the triceps (illustrated in the Figure 2B), $p>0.05$ between treatments (one-way ANOVA). (C)

Detection of laminin protein (green staining) by immunostaining on transverse sections of triceps from WT and *mdx* mice treated with saline, ASO or ASO+HDACi. Nuclei are labelled with dapi (blue staining). Scale bar, 100µm. (D) Relative expression of follistatin quantified by RT-qPCR in diaphragm of mdx mice treated with ASO+HDACi (Givinostat, valproic acid or EX527). N=7 mice per group, $p>0.05$ between treatments (one-way ANOVA). (E) Relative quantification of *Dmd* transcript levels obtained in diaphragm of mdx mice treated with ASO+HDACi (Givinostat, valproic acid or EX527) for several exon−exon junctions along the *Dmd* gene normalized to C57Bl10 mice, N=6 mice per group (two-way ANOVA).

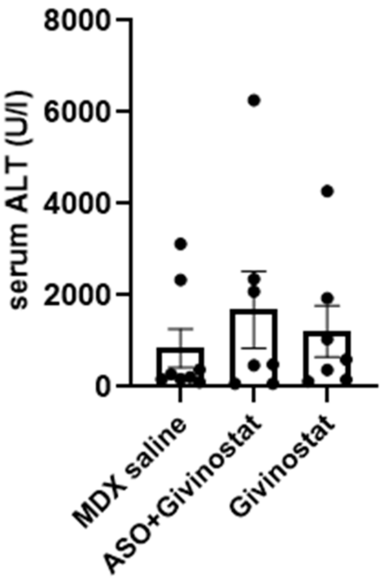

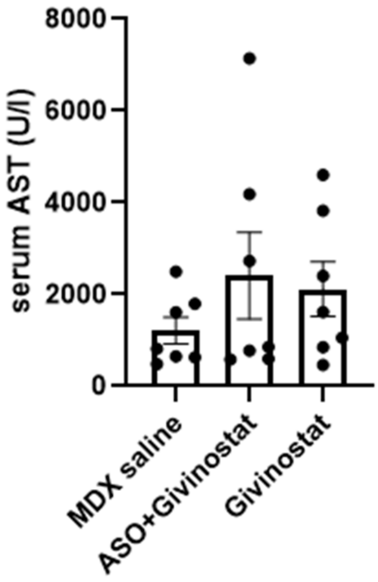

One way Anova (Kruskal-Wallis)	P=0.8047
Mann-Whitney saline vs ASO+Givi	P=0.6126
Mann-Whitney saline vs Givi	P=0.6126

One way Anova (Kruskal-Wallis)	P=0.5809
Mann-Whitney saline vs ASO+Givi	P=0.5350
Mann-Whitney saline vs Givi	P=0.3176

Figure S3: Effect of Givinostat on serum transaminases level

Quantification of transaminases in the serum of *mdx* mice treated with PBS (Saline), ASO+Givinostat or Givinostat alone: alanine aminotransferase (ALT), aspartate aminotransferase (AST), N=7 mice per group. Statistical analysis was performed using two different tests as reported in the tables: One-way Anova and Mann-Whitney test for the comparison two per two.

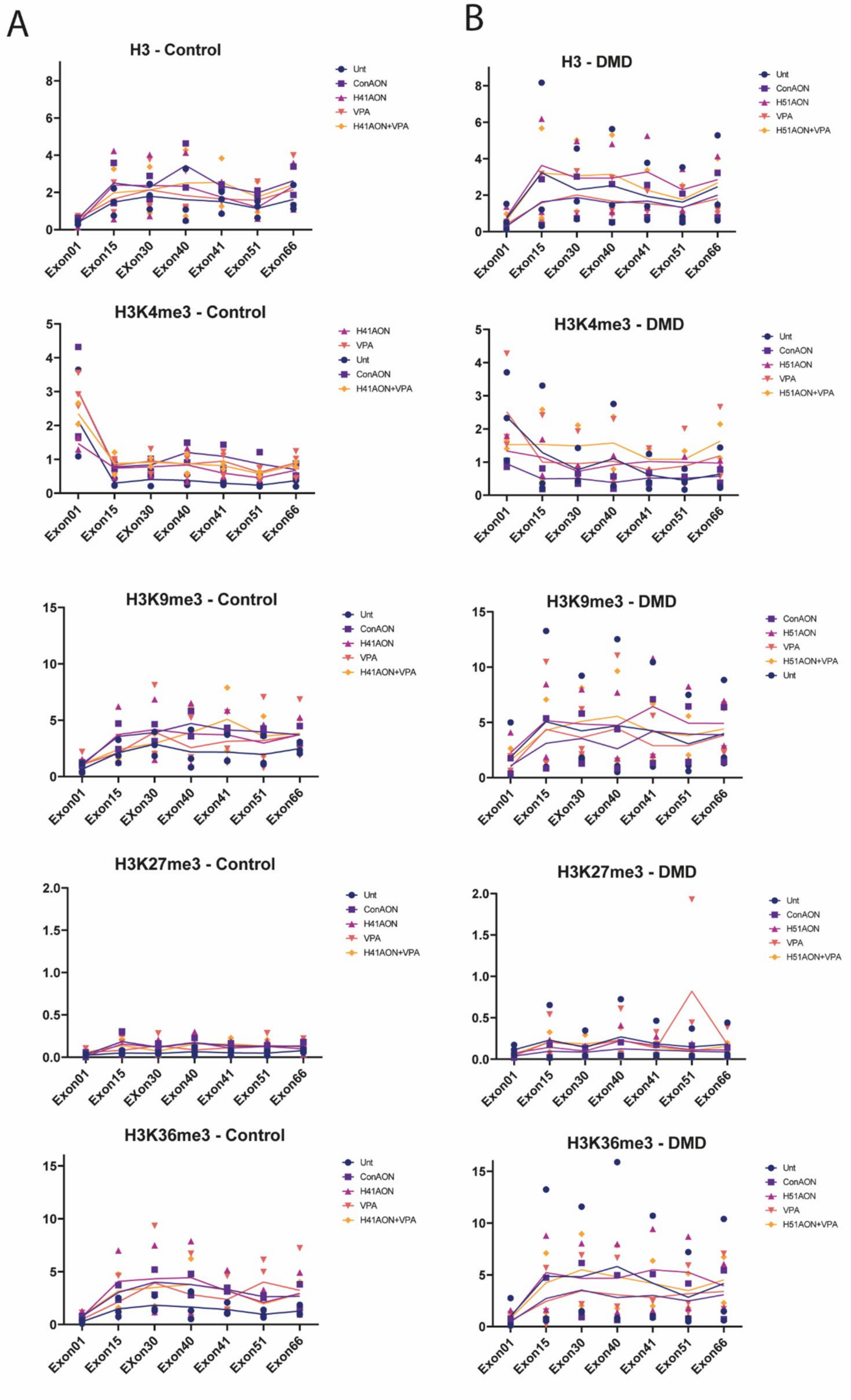
A
B
H3 - Control
H3K4me3 - Control
H3K9me3 - Control
H3K27me3 - Control
H3K36me3 - Control
H3 - DMD
H3K4me3 - DMD
H3K9me3 - DMD
H3K27me3 - DMD
H3K36me3 - DMD
Unt
ConAON
H41AON
VPA
H41AON+VPA
H51AON
H51AON+VPA
Exon01
Exon15
EXon30
Exon30
Exon40
Exon41
Exon51
Exon66

Figure S4: Effect of valproic acid on chromatin organization

Additional ChIP-qPCR data of healthy control cells (A) or DMD-patient cells (B). ChIP-qPCR analysis of the histone marks H3, H3K4me3, H3K9me3, H3K27me3 and H3K36me3 at various locations in the *DMD* locus in immortalised myocytes are shown as indicated. Cells were untreated (Unt), treated with a control ASO (targeting NOTCH3 (Con)), ASOs inducing exon skipping of *DMD* exon 41 (H41), *DMD* exon 51 (H51), valproic acid (VPA), or a combination of ASOs and VPA as indicated. Enrichment of histone marks was normalized to input chromatin. N= 3 experiments.

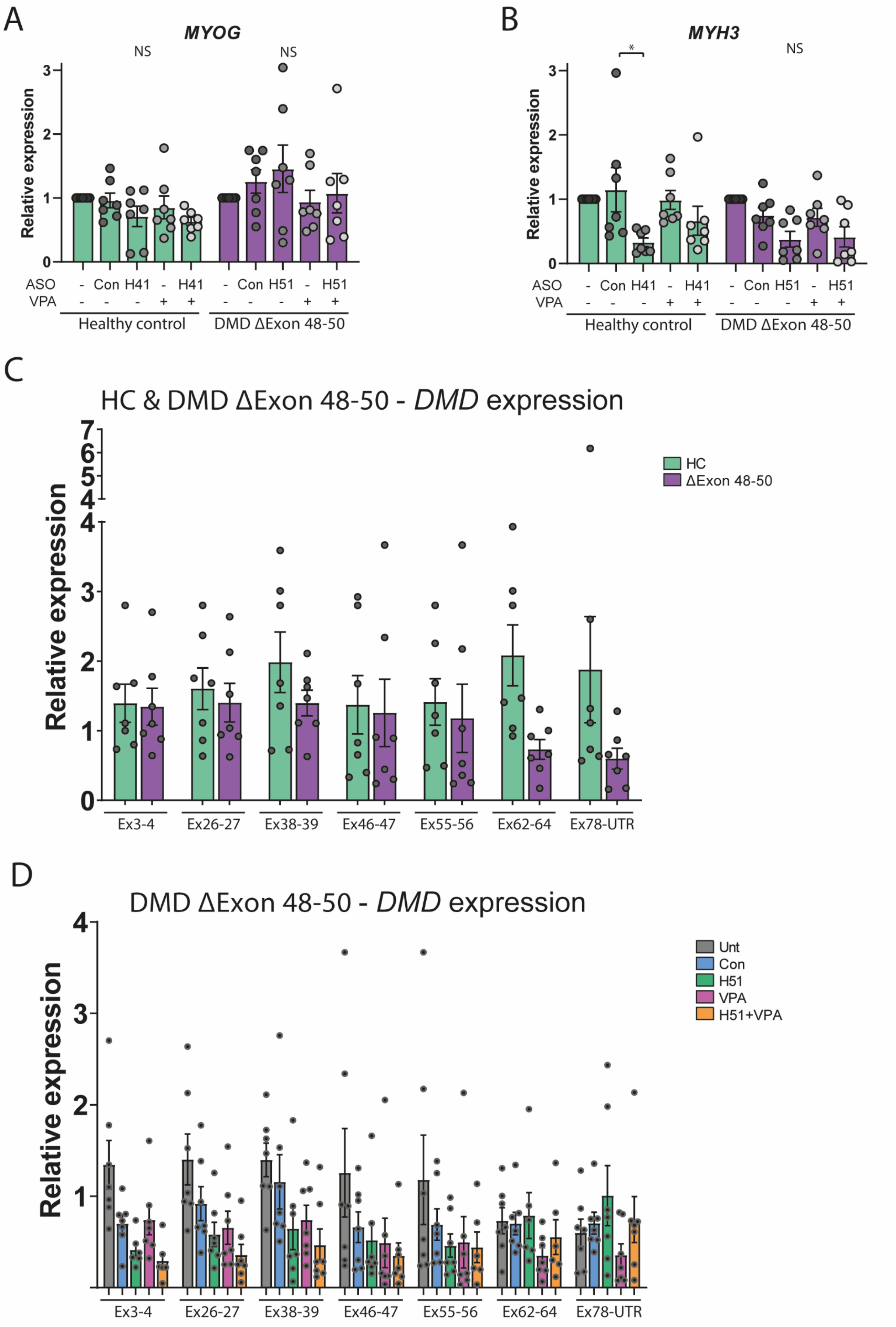
A
MYOG
NS
NS
Relative expression
ASO - Con H41 - H41 - Con H51 - H51
VPA - - - + + - - - + +
Healthy control
DMD ΔExon 48-50
B
MYH3
*
NS
Relative expression
ASO - Con H41 - H41 - Con H51 - H51
VPA - - - + + - - - + +
Healthy control
DMD ΔExon 48-50
C
HC & DMD ΔExon 48-50 - DMD expression
Relative expression
HC
ΔExon 48-50
Ex3-4 Ex26-27 Ex38-39 Ex46-47 Ex55-56 Ex62-64 Ex78-UTR
D
DMD ΔExon 48-50 - DMD expression
Relative expression
Unt
Con
H51
VPA
H51+VPA
Ex3-4 Ex26-27 Ex38-39 Ex46-47 Ex55-56 Ex62-64 Ex78-UTR

Figure S5: Myogenic potential and dystrophin RNA expression in DMD cells

(A) RT-qPCR analysis of the expression of *MYOG* (A) or *MYH3* (B) in myotube samples corresponding to the samples presented in figures 5A and 5C. Gene expression was normalized to housekeeping genes *GUSB* and *GAPDH.* *: $P<0.05$, NS: Not-significant – Kruskal-Wallis test. (C) RT-qPCR data for various *DMD* exon junctions as indicated in healthy control and DMD patient cells. Gene expression was normalized to housekeeping genes *GUSB* and *GAPDH.* (D) RT-qPCR data for various *DMD* exon junctions as indicated in DMD-patient cells. Gene expression was normalized to housekeeping genes *GUSB* and *GAPDH.*

Table S1: primers used (RT-qPCR, RT-PCR and ChIP-qPCR)

Name:	Forward 5'-->3' Sequence:	Reverse 5'-->3' Sequence:	Gene symbol
RT-qPCR_cDNA-GUSB	CCGAGTGAAGATCCCCTTTTTA	CTCATTTGGAATTTTGCCGATT	GUSB
RT-qPCR_cDNA-GAPDH	CTCTGCTCCTCCTGTTCGAC	ACGACCAAATCCGTTGACTC	GAPDH
RT-qPCR_cDNA-MYOG	GCCAGACTATCCCCTTCCTC	GGGGATGCCCTCTCCTCTAA	MYOG
RT-qPCR_cDNA-MYH3	CCTGCTGGAGGTGAAGTCTC	GATTGCAGGATCTGGTGGAT	MYH3
RT-qPCR_cDNA DMD exon 03-04	gggaagcagcatattgagaac	gggcatgaactcttgtggat	DMD
RT-qPCR_cDNA DMD exon 26-27	aagatctatcagagatgcacg	gttgggcctcttctttagctct	DMD
RT-qPCR_cDNA DMD exon 38-39	attgcttgaaccactggagg	TTTACAGTACCCTCATTGTCTTCAT	DMD
RT-qPCR_cDNA DMD exon 46-47	gttttatggttggaggaagcagat	gagcacttacaagcacggg	DMD
RT-qPCR_cDNA DMD exon 55-56	caggatgctacccgtaagga	cgtctttgtaacaggactgc	DMD
RT-qPCR_cDNA DMD exon 62-64	gccaaacaaagtgccctacta	ctgagaatctgacattattcagg	DMD
RT-qPCR_cDNA DMD exon 78-UTR	cctggaaagccaatgagaga	gcgggaatcaggagttgtaa	DMD
RT-PCR - DMD Exon40-42	GGCTCTAGAAATTTCTCATCAGTG	ggcatgtcttcagtcatcac	
RT-PCR - DMD Exon47-52	cccataagcccagaagagc	tctagcctcttgattgctgg	
ChIP-qPCR - DMD_Exon1	AAGCTGCTGAAGTTTGTTGG	TCTTCCCACCAAAGCATTTT	
ChIP-qPCR - DMD_Exon15	cctttttagtgcatggctttc	GGCCAGTTTTTGAAGACTTGAT	
ChIP-qPCR - DMD_Exon30	AGTCTGCCCAGGAGACTGAA	CGTCCACCTTGTCTGCAATA	
ChIP-qPCR - DMD_Exon40	GGCTCTAGAAATTTCTCATCAGTG	ATTTTCCTTTCATCTCTGGGC	
ChIP-qPCR - DMD_Exon41	GATCGGGAATTGCAGAAGAA	ATCTGAGTTGGCTCCACTGC	
ChIP-qPCR - DMD_Exon51	ggcttggacagaacttaccg	cttctgcttgatgatcatctc	
ChIP-qPCR - DMD_Exon66	gaggatccgtgtcctgtctt	tttacacagggaaatgatgcc	

Table S2: Antibodies used

Antibody:	Supplier (Cat. Nr.)	Dil. WB	Dil. ChIP
Histone H3	Abcam - Ab1791	N/A	2.5 µl for 3 µg Chromatin
H3K4me3	Millipore - 17-614	N/A	3 µl for 3 µg Chromatin
H3K9me3	Abcam - Ab8898	N/A	1.5 µl for 3 µg Chromatin
H3K9Ac	Abcam - Ab4441	N/A	2 µg for 3 µg Chromatin
H3K27me3	Millipore - 17-622	N/A	4 µl for 3 µg Chromatin
H3K36me3	Diagenode - C15410058	N/A	2.2 µl for 3 µg Chromatin
IgG control	Cell signaling - 2729S	N/A	1.5 µl for 3 µg Chromatin
Rabbit anti Dystrophin	Abcam - Ab154168	1:2000	N/A
Mouse anti Tubulin	Sigma-Aldrich - T6199	1:5000	N/A
N/A: Not applied for technique.			
